# FOSL1-PRMT1 transcriptional-epigenetic circuit promotes glioblastoma radioresistance via calcyphosine-mediated DNA repair and invasion

**DOI:** 10.1186/s43556-025-00394-0

**Published:** 2025-12-22

**Authors:** Yating Zhang, Jiajia Tian, Shuai Wu, Yiting Zhou, Zhongyuan Bao, Yi Zhu, Peng Wang, Zixiang Liu, Pengpeng Li, Zhenxing Tao, Zengli Miao, Xiaojie Lu, Xudong Zhao

**Affiliations:** 1 Neuroscience Center, Wuxi School of Medicine, Jiangnan University, Wuxi, Jiangsu Province 214122 China; 2 Department of Neurosurgery, Jiangnan University Medical Center, Wuxi, Jiangsu Province 214122 China; 3 Department of Neurosurgery, Wuxi No. 2 People’s Hospital, Affiliated Wuxi Clinical College of Nantong University, Wuxi, 214002 China; 4 Department of Intervention Therapy, The Affiliated Hospital of Jiangnan University, Wuxi, 214002 China; 5 Wuxi Neurosurgical Institute, Wuxi, 214002 China

**Keywords:** FOSL1, PRMT1, CAPS, Glioblastoma, DNA damage repair, Radioresistance

## Abstract

Radioresistance constitutes a major therapeutic barrier in glioblastoma (GBM), frequently leading to tumor recurrence and poor clinical outcomes. Despite advances in multimodal therapies, the molecular mechanisms underlying this resistance remain incompletely understood, limiting the development of effective interventions. This study identifies Fos-like antigen 1 (FOSL1) as a key driver of therapy resistance, with its elevated expression linked to poor prognosis in recurrent GBM. Functionally, FOSL1 knockdown sensitizes GBM cells to irradiation, impairs DNA damage repair, and reduces cell invasiveness. Mechanistically, FOSL1 physically interacts with and stabilizes Protein Arginine Methyltransferase 1 (PRMT1). This interaction enhances PRMT1-mediated asymmetric dimethylation of histone H4 (H4R3me2a) and facilitates methylation of Poly(A) Binding Protein Nuclear 1 (PABPN1). PRMT1, in turn, transcriptionally upregulates Calcyphosine (CAPS), which is essential for the pro-resistance and pro-invasive functions of this axis. We further demonstrate that the FOSL1-PRMT1-CAPS axis concurrently activates both Homologous Recombination (HR) and Non-Homologous End Joining (NHEJ) repair pathways to promote therapeutic resistance. In an orthotopic GBM mouse model, genetic or pharmacological disruption of this axis significantly enhances radiosensitivity and suppresses tumor invasion. Collectively, these findings unveil a previously unrecognized signaling pathway that coordinately regulates DNA repair fidelity and invasive potential in GBM. Our work proposes the FOSL1‑PRMT1‑CAPS axis as a promising therapeutic target for overcoming radioresistance and improving treatment outcomes in GBM patients.

**Supplementary Information:**

The online version contains supplementary material available at 10.1186/s43556-025-00394-0.

## Introduction

Glioblastoma (GBM) represents a critical clinical challenge, accounting for approximately 14–16% of all primary brain tumors and 48–50% of malignant primary brain tumors in adults [1, 2]. Despite this prevalence, the 5-year survival rate remains devastatingly low [3, 4]. Although radiotherapy serves as the cornerstone of GBM treatment [5], the development of radioresistance frequently leads to tumor recurrence and patient death, posing a major therapeutic obstacle [6]. Therefore, elucidating the molecular mechanisms of radioresistance and identifying novel therapeutic targets are urgently needed to improve treatment outcomes [7, 8]. Ionizing radiation (IR) induces a spectrum of DNA lesions, with DNA double-strand breaks (DSBs) representing the most critical and deleterious form, activating complex DNA damage response (DDR) pathways [9, 10]. Homologous recombination (HR) and non-homologous end joining (NHEJ) are the predominant mechanisms responsible for repairing IR-induced DSBs [11]. In tumor cells, hyperactivation of these DDR pathways is a key mechanism of radioresistance, facilitating efficient DNA repair and promoting cell survival [12–14]. Consequently, targeting HR and NHEJ components has emerged as a promising radiosensitization strategy, with considerable advances achieved in recent years [15]. The FOS family, core components of the activator protein-1 (AP-1) transcription factor complex, plays a pivotal role in oncogenesis and gene expression dysregulation [16, 17]. Among them, Fos-like antigen 1 (FOSL1) has gained attention for its prognostic significance across multiple cancers, where its elevated expression correlates with tumor progression and poor patient survival [18]. FOSL1 also promotes epithelial-mesenchymal transition (EMT), thereby facilitating cancer invasion and metastasis [19]. However, the role of FOSL1 in modulating GBM radiosensitivity remains unexplored.

The enzymatic activity of protein arginine methyltransferases (PRMTs) installs arginine methylation, a post-translational modification with a well-established central role in fundamental processes including transcriptional regulation, DNA repair, and signal transduction [20, 21]. This modification is also a major driver of cancer development and metastasis [22–24]. Protein Arginine Methyltransferase 1 (PRMT1), the predominant type I PRMT, catalyzes asymmetric dimethylarginine (ADMA) on diverse protein substrates [23]. Mounting evidence indicates that PRMT1-mediated ADMA modification on nonhistone targets not only influences basic cellular machinery but also directly contributes to oncogenesis and metastatic dissemination [25–27].

Calcyphosine (CAPS) is an EF-hand domain-containing calcium-binding protein implicated in calcium-phosphatidylinositol and cyclic AMP (cAMP)-mediated signaling pathways, through which it regulates a variety of cellular functions [28]. Although CAPS overexpression has been reported in multiple cancer types—including ependymoma [29], endometrial [30], lung [31] and colorectal [32], its functional significance in glioma remains incompletely understood. Previous experimental evidence indicates that elevated CAPS expression enhances glioma cell proliferation in both in vitro and in vivo settings [33], suggesting a previously underestimated oncogenic function in glioma pathogenesis.

In this study, we confirmed FOSL1 expression and explored its regulatory role in GBM proliferation and metastasis. The primary focus of our research was to ascertain the role of FOSL1 in radioresistance and dig deeper into the underlying mechanisms implicated in GBM. Furthermore, we established that FOSL1 has the capacity to interact with and activate PRMT1, thus elevating the expression of CAPS and inducing radioresistance and the invasion of GBM. Our study provides evidence that targeting FOSL1 represents a promising therapy for GBM.

## Results

### FOSL1 is identified as a key factor linked to radioresistance in GBM

**Fig. 1:**
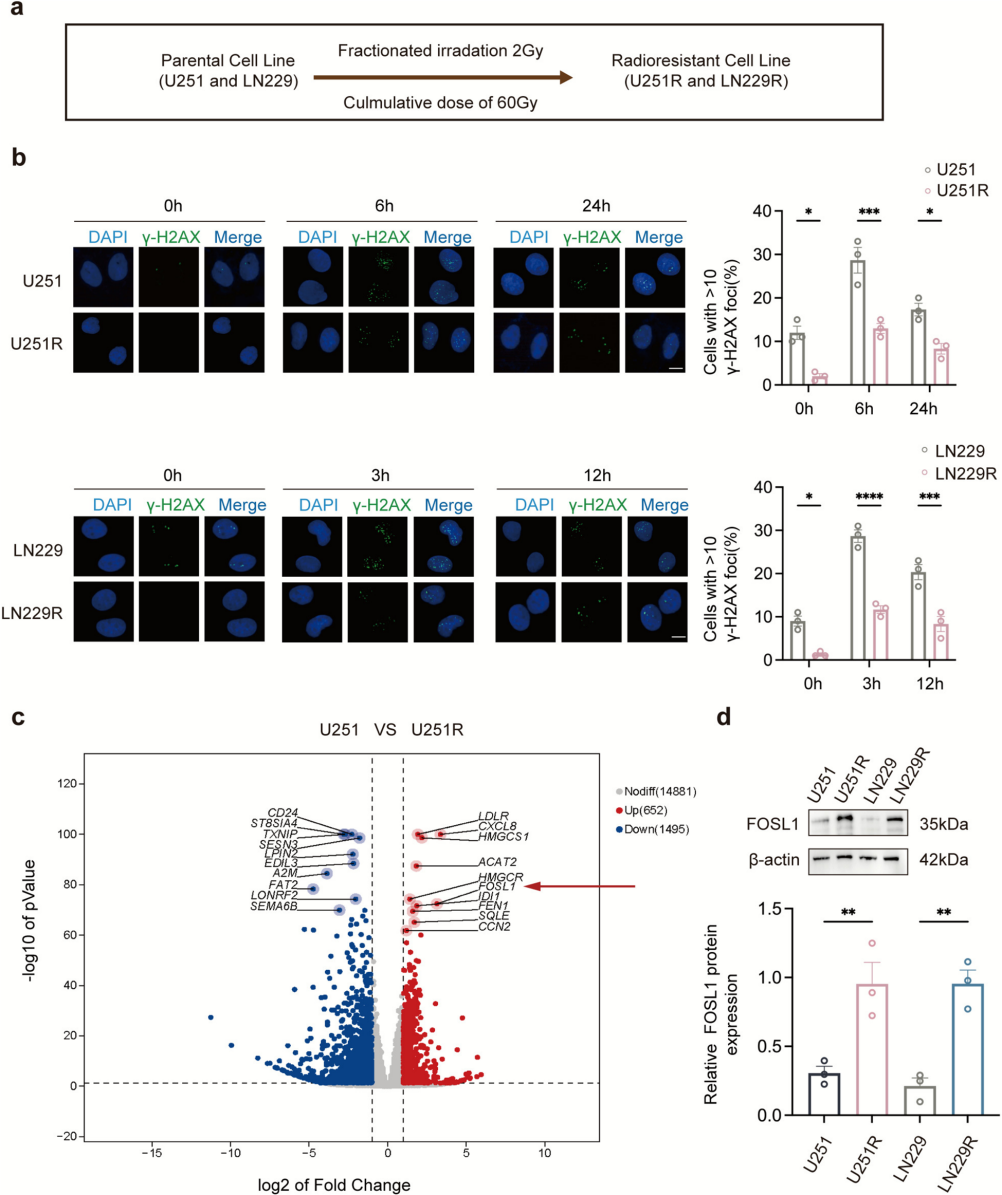
FOSL1 correlates negatively with GBM radiosensitivity. **a** Scheme for generating radioresistant (U251R, LN229R) cells. **b** γ-H2AX foci (green) in parental and radioresistant cells at indicated times post-IR. Nuclei (blue, DAPI). Scale bar: 20 μm. **c** Volcano plot of DEGs in U251R vs. U251. **d** Western blot and quantitation of FOSL1 in cell lines. *p < 0.05, **p < 0.01, ***p < 0.001, ****p < 0.0001

**Fig. 2:**
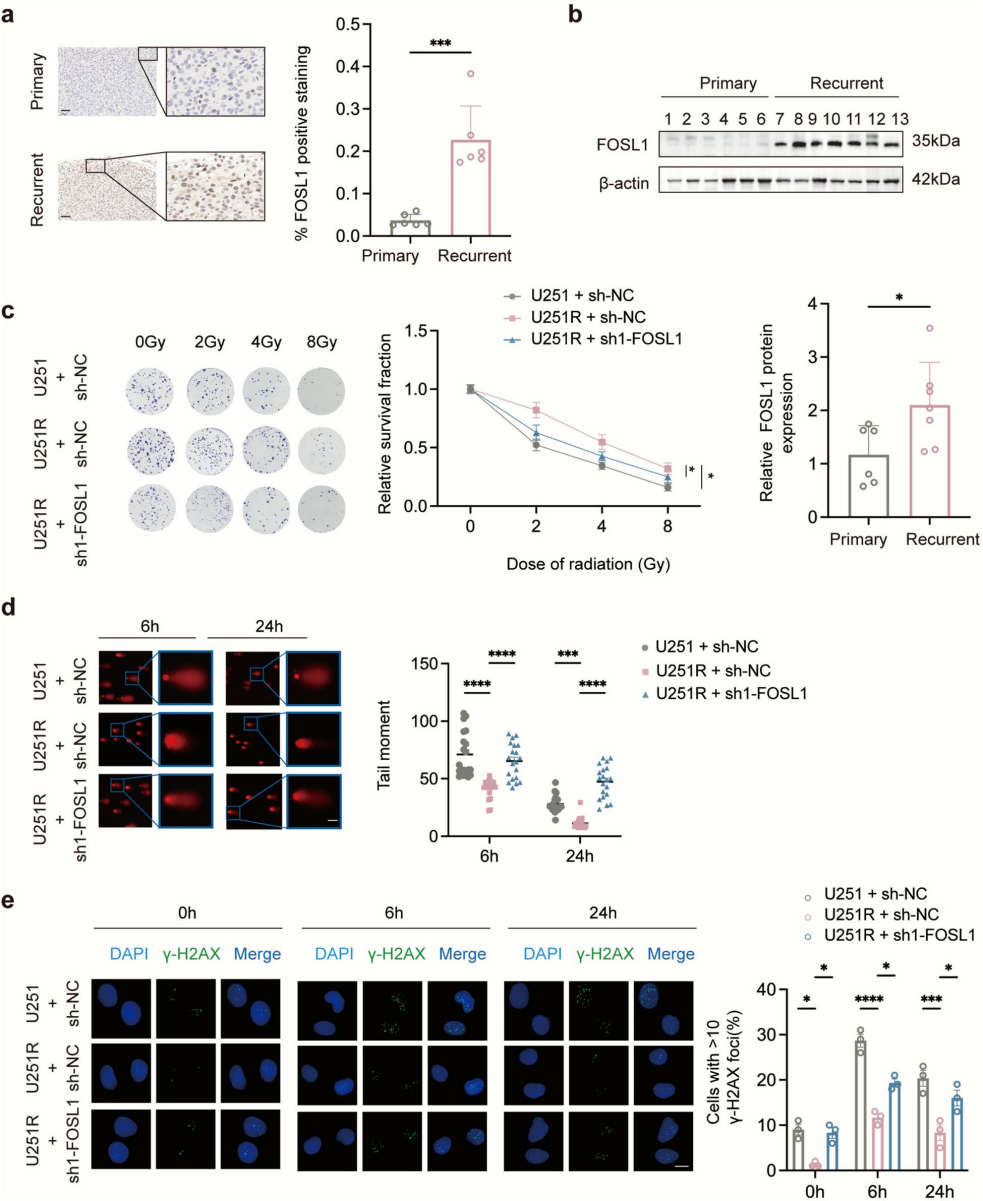
FOSL1 promotes radioresistance in GBM by modulating the repair of irradiation-induced DSBs.** a** IHC staining of FOSL1 in primary and recurrent GBM tissues (*n* = 6 per group). Scale bar: 50 μm. **b** Western blot analysis of FOSL1 protein levels in primary (*n* = 6) and recurrent (*n* = 7) GBM tissues. **c** Colony formation assays of control and FOSL1-knockdown cells after exposure to the indicated doses of irradiation. **d** Comet assays and **e** representative immunofluorescence images with quantification of γ-H2AX foci in control and FOSL1-knockdown cells at the indicated times post-IR. Scale bars: 20 μm (**d, e**). *p < 0.05, ***p < 0.001, ****p < 0.0001

To investigate the molecular basis of radiation resistance in GBM, we established radioresistant LN229R and U251R cell lines by exposing the parental LN229 and U251 cells to fractionated X-ray irradiation (Fig. 1a) [34]. The significantly enhanced radioresistance of these cells was confirmed using the CCK-8 assay (Fig. S1a). IR induces DSBs, which are marked by the phosphorylation of histone H2AX at serine 139 (γ-H2AX) at damage sites. Consequently, γ-H2AX serves as a reliable biomarker for DSBs and is linked to radiosensitivity [26]. We next evaluated γ-H2AX levels in GBM cells following IR. The number of γ-H2AX-positive nuclei increased sharply immediately after IR and then gradually decreased over time. As shown in Fig. 1b, LN229R and U251R cells displayed a reduced number of γ-H2AX foci compared to the control cells. To identify genes associated with radioresistance in GBM, we performed RNA sequencing to compare the transcriptional profiles of U251 and U251R cell lines. This analysis identified a set of differentially expressed genes, with FOSL1 being notably upregulated (Fig. 1c). Clinically, Kaplan–Meier survival analysis indicated that high FOSL1 expression was correlated with a poorer prognosis in GBM patients (Fig. S2a). In contrast, the other top differentially expressed genes did not show a significant association with survival outcomes to the extent that FOSL1 did (Fig. S3). Based on these findings, we selected FOSL1 for further functional investigation. Both western blot analysis (Fig. 1d) and Real-time quantitative PCR (RT-qPCR) (Fig. S1b) revealed a clear upregulation of FOSL1 in radioresistant cells compared to their parental counterparts. To corroborate the pattern of FOSL1 expression in GBM patients, we performed immunohistochemistry (IHC) staining on primary and recurrent GBM tissues. The results showed FOSL1 protein overexpression in recurrent GBM tissues (Fig. 2a). Additionally, western blot analysis of six primary and seven recurrent GBM tissues demonstrated higher levels of FOSL1 protein in recurrent tumors than in primary tumors (Fig. 2b). Collectively, these findings suggest that FOSL1 may play a role in tumor progression and radioresistance in GBM.

### FOSL1 knockdown sensitizes GBM cells to irradiation

To investigate the role of FOSL1 in IR-induced DNA damage in GBM cells, we knocked down FOSL1 expression and confirmed the efficiency by western blotting (Fig. S1c). Since sh1-FOSL1 demonstrated the strongest knockdown efficacy, it was selected for all subsequent experiments (Fig. S1d, e). We then assessed the effect of FOSL1 on radioresistance using colony formation assays. The results showed that FOSL1 knockdown significantly reduced the clonogenic survival of GBM cells across a range of radiation doses, indicating that FOSL1 enhances radioresistance (Fig. 2c, S4a). To directly quantify DNA damage, we performed comet assays after 4 Gy IR exposure. FOSL1-deficient cells exhibited a significantly higher olive tail moment than control cells (Fig. 2d, S4b). Consistent with this, immunofluorescence staining revealed that the percentage of γ-H2AX-positive cells was markedly increased upon FOSL1 knockdown following IR (Fig. 2e, S4c). Collectively, these data demonstrate that FOSL1 confers radioresistance to GBM cells.

### FOSL1 promotes radioresistance by enhancing invasiveness in GBM

**Fig. 3:**
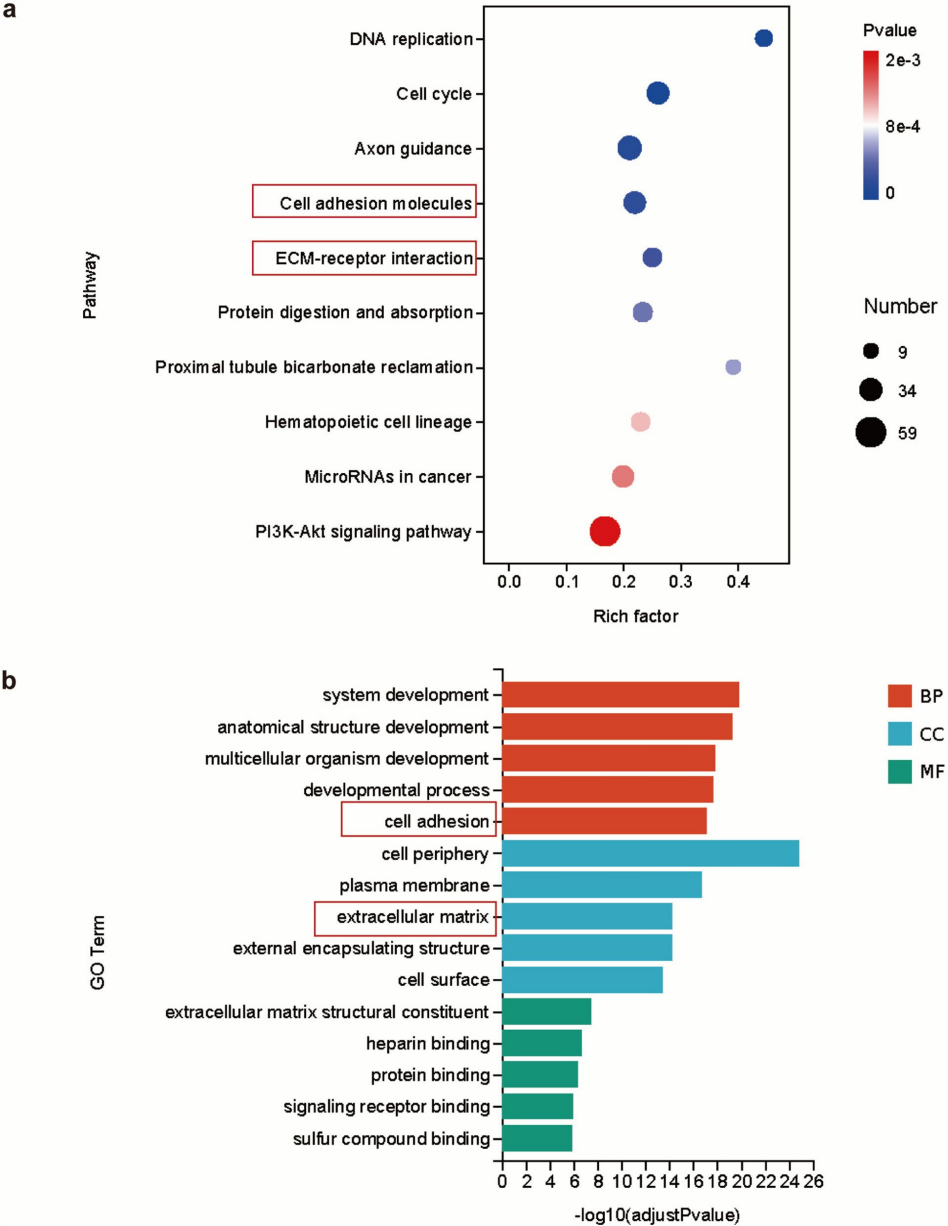
Pathways associated with cell invasion. **a**, **b** KEGG and GO enrichment analysis of transcriptomic data from U251 and U251R cells

Transcriptomic sequencing combined with KEGG and GO enrichment analyses revealed a significant enrichment of cell adhesion pathways in U251R cells compared to U251 cells (Fig. 3a, b), a process closely associated with tumor invasiveness. To investigate how FOSL1 promotes invasion, we analyzed key molecular markers of the EMT and extracellular matrix (ECM) remodeling—specifically, Zonula occludens-1 (ZO-1), Neural-cadherin (N-cadherin), and Matrix Metalloproteinase-2 (MMP2)—under both baseline and irradiated conditions. IR upregulated the expression of these proteins, whereas FOSL1 knockdown significantly suppressed their levels (Fig. S4d). These results suggest that FOSL1 facilitates GBM progression by augmenting EMT-like processes and ECM degradation. Collectively, our data reinforce the oncogenic role of FOSL1 and indicate that its contribution to radioresistance may be partially mediated through the enhancement of invasive capability.

### The FOSL1-PRMT1 axis regulates H4R3me2a and PABPN1 methylation

**Fig. 4:**
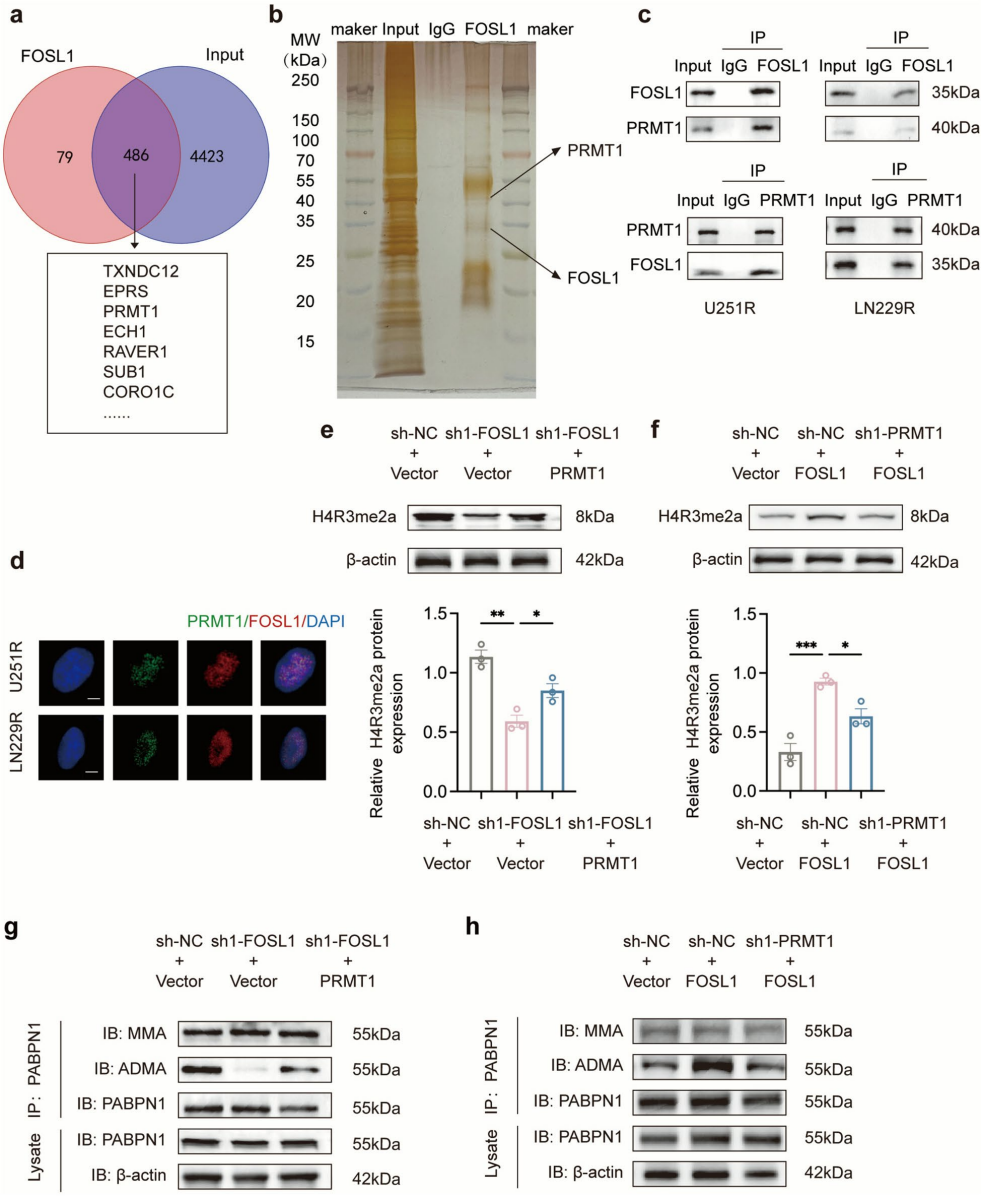
FOSL1 interacts with and activates PRMT1. **a** Venn diagram illustrating the overlap between the input and anti-FOSL1 immunoprecipitation groups from RNA-seq analysis of U251R cells. **b** Silver staining of proteins co-immunoprecipitated with FOSL1. **c** Endogenous interaction between FOSL1 and PRMT1 was confirmed by reciprocal co-immunoprecipitation (co-IP) using the indicated antibodies in U251R and LN229R cell lysates. **d** Confocal microscopy images showing nuclear colocalization of PRMT1 (green) and FOSL1 (red) in U251R and LN229R cells. Nuclei were stained with DAPI (blue). Scale bar: 10 μm. **e, f** Western blot analysis of H4R3me2a levels under the indicated conditions. **g, h** IP and immunoblot (IB) analysis of PABPN1 MMA and ADMA under the indicated conditions. *p < 0.05, **p < 0.01, ***p < 0.001, ****p < 0.0001

To elucidate the mechanism by which FOSL1 modulates radioresistance in GBM cells, we conducted an immunoprecipitation (IP) experiment. A Venn diagram illustrates the overlap between the input and anti-FOSL1 immunoprecipitated groups in U251R cells (Fig. 4a). Given that PRMT1 promotes radiotherapy resistance in glioma and was identified as a potential FOSL1 interactor, we hypothesized that FOSL1 interacts with PRMT1. Reciprocal co-IP assays confirmed this interaction in both U251R and LN229R cell lines (Fig. 4c), which was visualized by silver staining of the co-IP samples (Fig. 4b). Furthermore, immunofluorescence staining demonstrated clear nuclear colocalization of FOSL1 and PRMT1 in GBM cells (Fig. 4d). We found that FOSL1 knockdown reduced PRMT1 protein levels (Fig. S5b), whereas PRMT1 knockdown did not affect FOSL1 expression (Fig. S5c; PRMT1 knockdown efficiency is shown in Fig. S5a). These results suggest that FOSL1 acts upstream to regulate PRMT1.

As PRMT1 is the primary enzyme catalyzing asymmetric dimethylation of histone H4 at arginine 3 (H4R3me2a) [35], we investigated whether FOSL1 influences this activity. Western blot analysis showed that FOSL1 knockdown significantly reduced H4R3me2a levels, an effect rescued by PRMT1 overexpression. Conversely, FOSL1 overexpression increased H4R3me2a (Fig. 4e, f), demonstrating that FOSL1 regulates PRMT1-mediated H4R3me2a deposition.

Given that arginine methylation of Poly (A) Binding Protein Nuclear 1 (PABPN1) is a known PRMT1 substrate and its expression is associated with GBM aggressiveness [36, 37], we sought to determine whether it contributes directly to the radioresistant phenotype of GBM cells, a possibility that had not yet been experimentally tested. Manipulating FOSL1 and PRMT1 expression revealed that FOSL1 knockdown specifically reduced the ADMA modification of PABPN1, without affecting monomethylarginine (MMA) levels. This reduction was reversed by PRMT1 overexpression (Fig. 4g, h). These data indicate that the FOSL1-PRMT1 axis specifically regulates the ADMA methylation of PABPN1. Furthermore, we assessed global N6-methyladenosine (m⁶A) methyltransferase activity across experimental groups. As shown in Fig. S6a, c, FOSL1 knockdown significantly suppressed global m⁶A methyltransferase activity compared with the control group, while this suppression was effectively rescued by concurrent PRMT1 overexpression. Conversely, FOSL1 overexpression enhanced global m⁶A methyltransferase activity, and this enhancement was attenuated upon PRMT1 knockdown (Fig. S6b, d). Collectively, these results demonstrate that FOSL1 positively regulates global m⁶A methyltransferase activity in a PRMT1-dependent manner.

Methyltransferase-like 3 (METTL3), a key RNA methyltransferase, plays a critical role in regulating tumor proliferation, migration, invasion, and progression[38]. We hypothesized that FOSL1 and PRMT1 may modulate METTL3 expression at the transcriptional level, leading to altered METTL3 protein levels and subsequent global changes in m⁶A modification. Western blot analysis confirmed that METTL3 protein expression is influenced by both FOSL1 and PRMT1 (Fig. S7a-d). To further investigate the underlying mechanism, we performed METTL3 knockdown under conditions of FOSL1 or PRMT1 manipulation. We assessed the m⁶A enrichment on target mRNAs. Consistent with previous reports identifying Poly (ADP-ribose) polymerase *(PARP1)*, Sirtuin 1 *(SIRT1)*, Tuberous sclerosis complex 1*(TSC1)*, Interferon regulatory factor 4 *(IRF4)*, and Synaptosome associated protein 29 *(SNAP29)* as METTL3 targets [38–42], we observed that METTL3 depletion reduced m⁶A enrichment on *PARP1*, *SIRT*1, *TSC1*, and *IRF4* transcripts, while increasing m⁶A enrichment on *SNAP29* mRNA (Fig. S8a, b).

### The PRMT1-CAPS axis promotes radioresistance by enhancing invasiveness in GBM

**Fig. 5:**
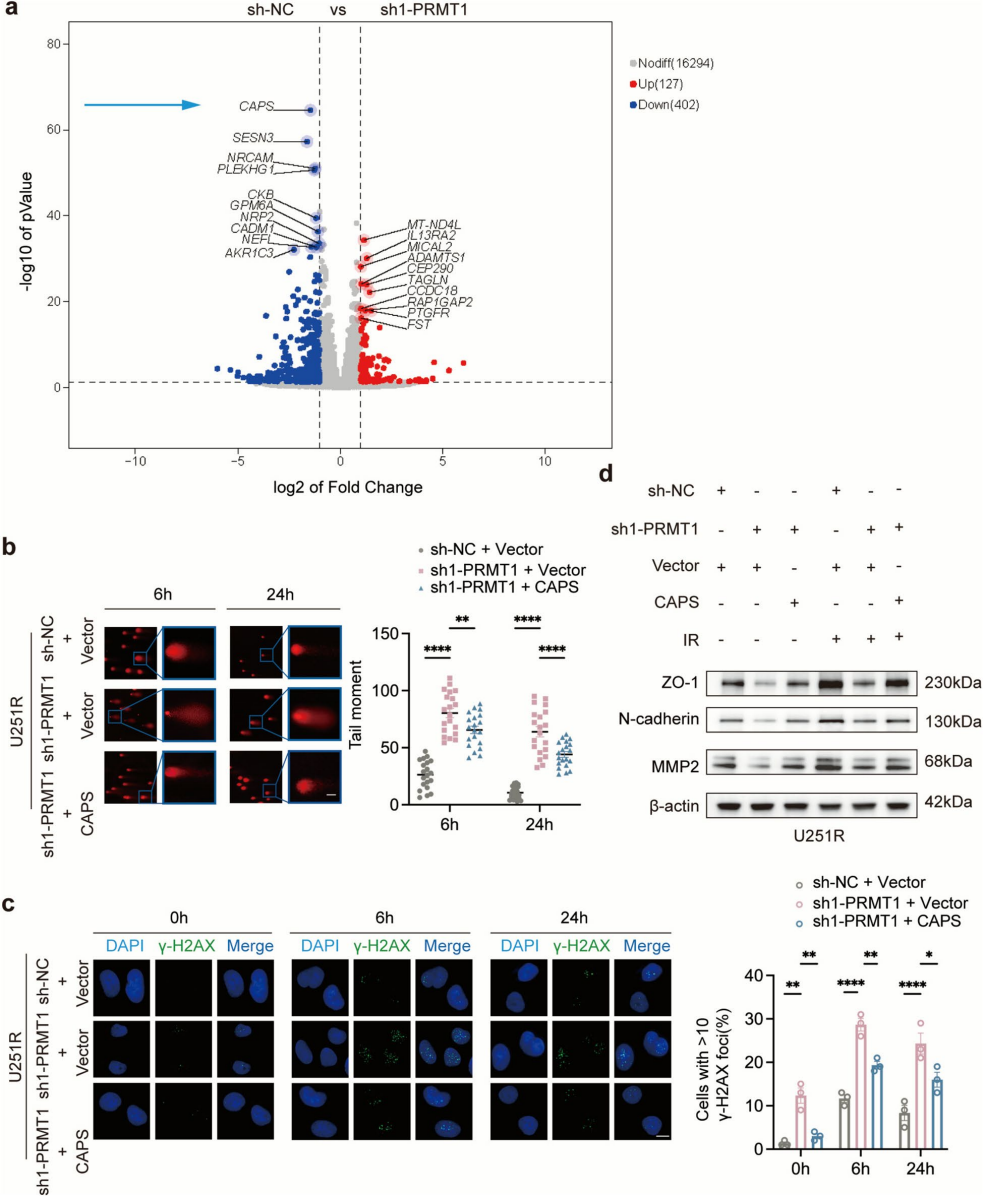
The PRMT1-CAPS axis promotes radioresistance in GBM by enhancing DNA repair and invasiveness. **a** Volcano plot of differentially expressed genes in U251R-sh1-PRMT1 cells compared to U251R-sh-NC control cells. **b** Comet assays assessing DNA damage in U251R cells under the indicated treatment conditions after 4 Gy irradiation. Quantification of the olive tail moment is shown in the adjacent panel. Scale bar: 20 μm. **c** Immunofluorescence analysis of γ-H2AX foci (green) in U251R cells under the indicated treatment conditions after 4 Gy irradiation. Nuclei were counterstained with DAPI (blue). Scale bar: 20 μm. **d** Western blot analysis of ZO-1, N-cadherin, and MMP2 protein levels in U251R cells under non-irradiated and 4 Gy irradiated conditions, following treatment with control vector, PRMT1 knockdown, or PRMT1 knockdown combined with CAPS overexpression. *p < 0.05, **p < 0.01, ***p < 0.001, ****p < 0.0001

RNA sequencing of control (sh-NC) and PRMT1-knockdown (sh1-PRMT1) cells revealed differentially expressed genes, among which CAPS was the most significantly downregulated candidate (Fig. 5a). This finding was independently validated by RT-qPCR (Fig. S5d, e). Given its pronounced upregulation and emerging relevance in cancer biology [33], CAPS was selected for further mechanistic study. Western blot analysis showed that PRMT1 knockdown reduced CAPS protein levels (Fig. S9a), then, CAPS expression was knocked down, with the efficiency confirmed by western blotting (Fig. S9b). Whereas CAPS perturbation did not affect PRMT1 expression (Fig. S9c), suggesting that CAPS acts downstream of PRMT1. To determine whether CAPS is involved in PRMT1-mediated DNA damage repair, we restored CAPS expression in PRMT1-deficient cells and confirmed reconstitution by western blot (Fig. S9d, e). Comet assays performed after 4 Gy irradiation showed that PRMT1 knockdown increased the olive tail moment, indicative of enhanced DNA damage, and this effect was reversed by CAPS overexpression (Fig. 5b, S10a). Consistent with these results, immunofluorescence staining demonstrated that PRMT1 depletion increased the number of γ-H2AX foci post-IR, and this phenotype was similarly rescued by CAPS reconstitution (Fig. 5c, S10b). These data collectively indicate that PRMT1 facilitates DNA damage repair in GBM through CAPS activation. At the molecular level, IR upregulated the expression of the invasion-related markers ZO-1, N-cadherin, and MMP2. PRMT1 knockdown suppressed their expression, while concurrent CAPS overexpression restored protein levels of all three markers (Fig. 5d, S10c). These findings suggest that the PRMT1-CAPS axis promotes GBM progression by inducing EMT-like changes and ECM degradation. Together, our results indicate that CAPS contributes to radioresistance in part by enhancing the invasive properties of GBM cells.

### The FOSL1-PRMT1-CAPS axis confers radioresistance through enhanced invasiveness in GBM

**Fig. 6:**
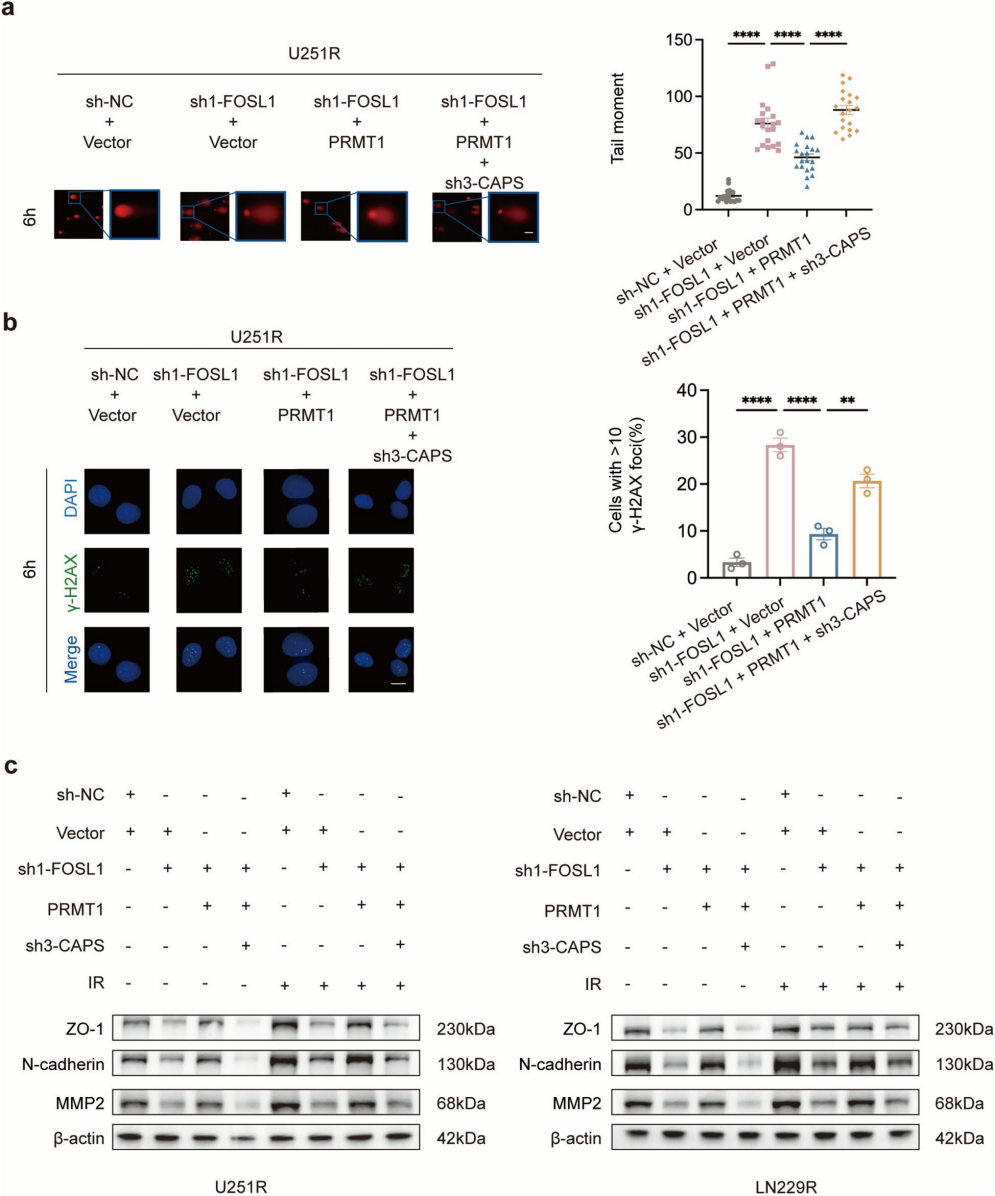
The FOSL1-PRMT1-CAPS axis confers radioresistance through enhanced invasiveness in GBM. **a** Comet assays assessing DNA damage in U251R cells under the indicated treatment conditions after 4 Gy irradiation. Scale bar: 20 μm. **b** Representative immunofluorescence images of γ-H2AX foci (green) in U251R cells under the indicated treatment conditions after 4 Gy irradiation. Nuclei were counterstained with DAPI (blue). Scale bar: 20 μm. **c** Western blot analysis of ZO-1, N-cadherin and MMP2 protein levels under the indicated treatment conditions. *p < 0.05, **p < 0.01, ***p < 0.001, ****p < 0.0001

Prompted by the association of high PRMT1 and CAPS expression with poor survival in GBM patients (Fig. S2b,c), and based on the molecular evidence of a FOSL1-PRMT1 interaction and PRMT1-mediated regulation of CAPS, we sought to determine whether these components function as a coordinated axis to promote radioresistance and invasiveness. First, U251R and LN229R cells were co-transfected with sh-NC, sh1-FOSL1, pcDNA3.1(+)-PRMT1 or empty vector, together with sh3-CAPS, and protein levels were analyzed by western blotting (Fig. S11a, b). Comet assays further showed that FOSL1 knockdown significantly increased the tail moment compared to the sh-NC group. This effect was rescued by PRMT1 overexpression, and then reversed again by CAPS knockdown (Fig. 6a, S12a). Immunofluorescence staining revealed that under 4 Gy irradiation, FOSL1 knockdown increased the formation of γ-H2AX foci, which was reversed by PRMT1 overexpression; however, subsequent knockdown of CAPS in this context induced γ-H2AX foci formation (Fig. 6b, S12b). Together, these results indicate that the FOSL1-PRMT1-CAPS axis cooperatively promotes radioresistance.

To examine whether the FOSL1-PRMT1-CAPS axis contributes to radioresistance partly by enhancing the invasive properties of GBM cells, we performed western blot analysis under both irradiated (4 Gy) and non-irradiated conditions. IR upregulated the expression of the invasion-related markers ZO-1, N-cadherin, and MMP2. Moreover, the expression levels of FOSL1, PRMT1, and CAPS were positively correlated with those of these invasion-related proteins (Fig. 6c). These findings suggest that the FOSL1-PRMT1-CAPS axis promotes GBM progression by inducing EMT-like changes and ECM degradation. In summary, our results demonstrate that the FOSL1-PRMT1-CAPS axis contributes to radioresistance in part by enhancing the invasive properties of GBM cells.

### FOSL1-PRMT1-CAPS axis modulates DDR through coordinated regulation of both HR and NHEJ pathways

**Fig. 7:**
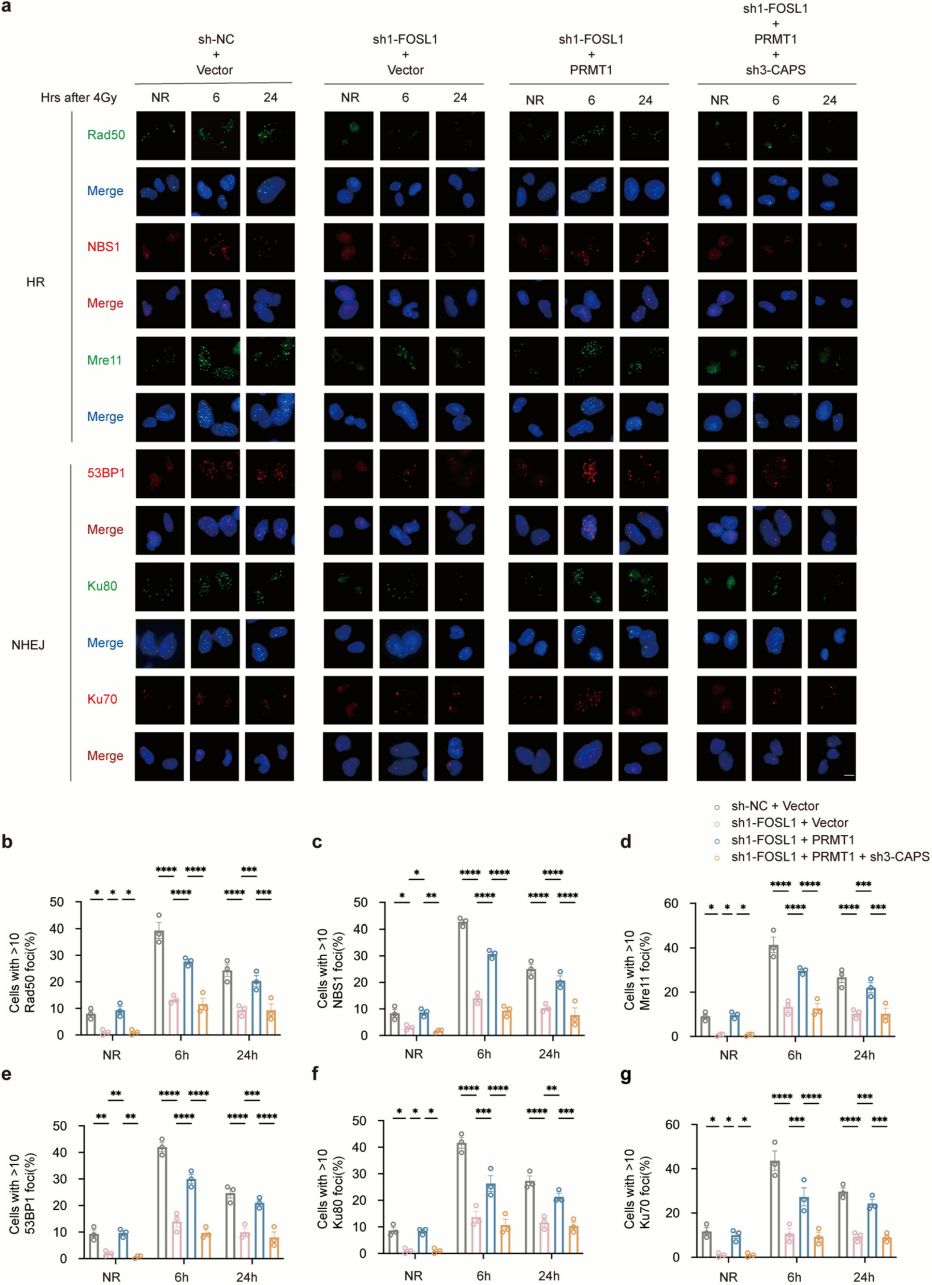
FOSL1-PRMT1-CAPS axis modulates DDR.** a** Representative immunofluorescence images of Rad50, Mre11, Ku80 foci (green) and 53BP1, NBS1, Ku70 foci (red) in U251R cells under the indicated treatment conditions after 4 Gy irradiation. Nuclei were counterstained with DAPI (blue). Scale bar: 20 μm. **b-g** Quantification of foci per cell. *p < 0.05, **p < 0.01, ***p < 0.001, ****p < 0.0001

HR and NHEJ represent two major DNA double-strand break repair pathways within the DDR network. To define the specific mechanisms through which the FOSL1-PRMT1-CAPS axis influences DDR, we performed immunofluorescence staining to monitor the dynamics of key repair proteins. The formation of nuclear foci by HR-associated factors (Rad50, NBS1, Mre11) and NHEJ-related components (53BP1, Ku80, Ku70) was tracked at various time points following 4 Gy irradiation (0 h serving as the unirradiated control). In general, the number of foci displayed a characteristic pattern, increasing initially and subsequently declining over time. Depletion of FOSL1 significantly reduced foci assembly across these markers; however, this suppression was effectively reversed by PRMT1 overexpression. Furthermore, additional knockdown of CAPS again attenuated foci formation, even in the presence of elevated PRMT1 (Fig. 7). Mechanistically, our findings reveal that the functional impact of the FOSL1-PRMT1-CAPS axis on DDR is mediated through its dual involvement in HR and NHEJ.

### The FOSL1-PRMT1-CAPS axis critically regulates radioresistance and invasiveness of GBM in vivo

**Fig. 8:**
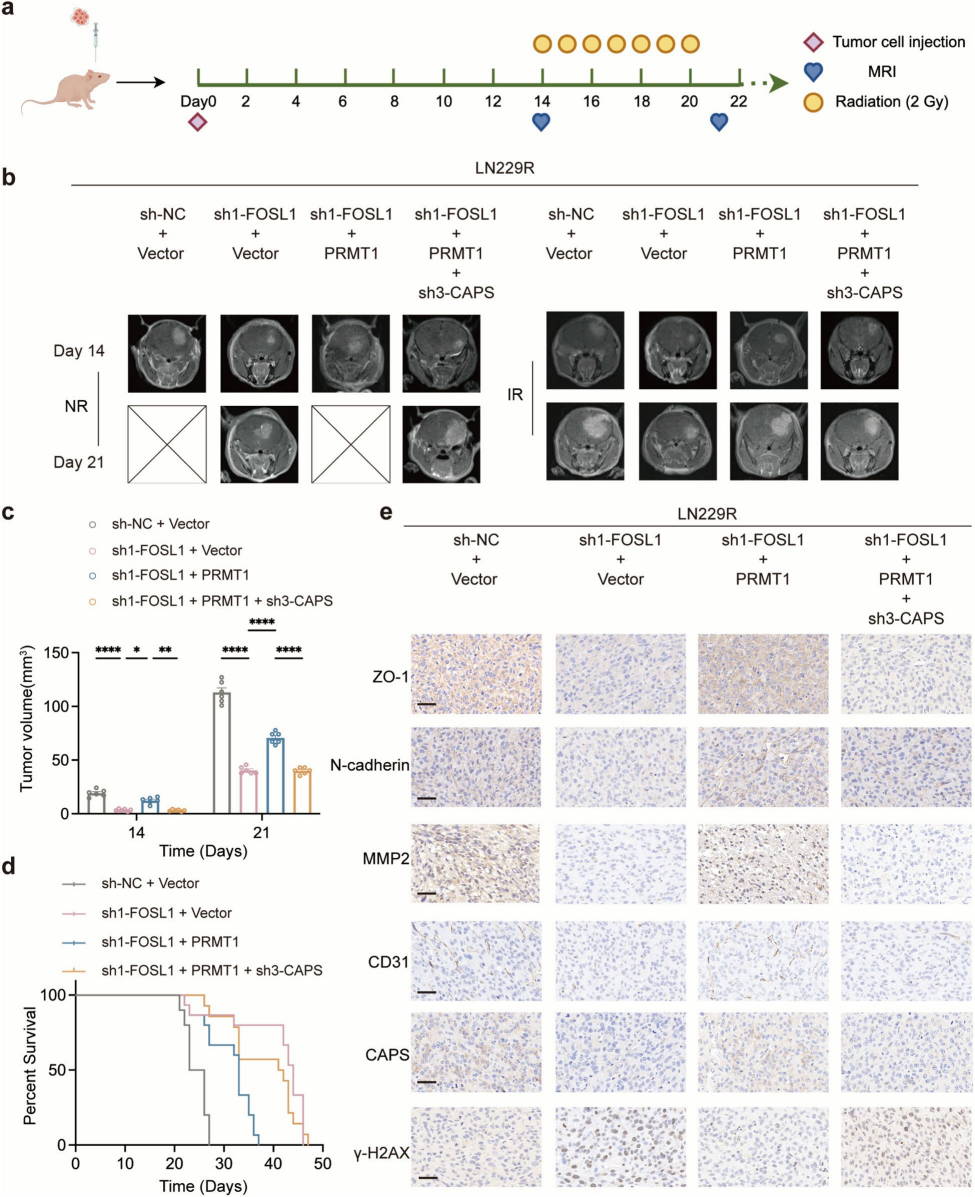
FOSL1-PRMT1-CAPS axis is associated with radioresistance and invasiveness of GBM in vivo. **a** Schematic illustration of the GBM orthotopic xenograft model. **b** MRI images of brain tumor growth. *n* = 6 for each group. **c** The brain tumor volume was quantified according to MRI images. **d** Kaplan–Meier survival curve of GBM mice. **e** IHC staining for ZO-1, N-cadherin, MMP2, CD31, CAPS and γ-H2AX in tumor samples. Scale bars, 20 μm. Data are represented as the mean ± SEM (*n* = 6). *p < 0.05; **p < 0.01; ***p < 0.001; **** p < 0.0001

To conduct a deeper exploration into the potential role of FOSL1-PRMT1-CAPS in enhancing radioresistance and invasiveness of GBM in vivo, an orthotopic mouse model of GBM was established through intracranial implantation. Nude mice were divided into four groups (n = 6) based on the injection of LN229R cells with different treatments. The four groups were the control group, FOSL1 knockdown group, FOSL1 knockdown with PRMT1 overexpression group, and FOSL1 knockdown and PRMT1 overexpression with CAPS knockdown group. Post intracranial tumor implantation, the animals were observed at 3-day intervals for alterations regarding their behavior and any decreases in body weight. MRI scans were conducted approximately on days 14 and 21 to evaluate tumor progression, during which time a radiation dose of 2 Gy/day was administered for 7 consecutive days (Fig. 8a). The MRI results indicated that the LN229R group with FOSL1 knockdown exhibited notably slower tumor growth compared to the LN229R control group. In contrast, the LN229R group with concurrent FOSL1 knockdown and PRMT1 overexpression demonstrated accelerated tumor progression in comparison to the group featuring FOSL1 knockdown, PRMT1 overexpression, and CAPS knockdown (Fig. 8b-d). IHC assay also suggested that the FOSL1-PRMT1-CAPS axis enhances the sensitivity to radiation and invasiveness of GBM cells in vivo (Fig. 8e).

## Discussion

Here we systematically explore the molecular mechanisms underlying radiotherapy resistance in GBM. Utilizing an integrated approach that combines radioresistant cell models with transcriptomic profiling, our findings point to a previously undercharacterized FOSL1-PRMT1-CAPS signaling axis as a critical contributor to therapeutic resistance in GBM. This work provides new insights into several aspects of GBM radioresistance. We identified FOSL1 as a bifunctional regulator that simultaneously enhances DNA repair capacity and promotes cellular invasion in irradiated GBM cells. Notably, our biochemical analyses demonstrate that direct FOSL1-PRMT1 binding enhances PRMT1's methyltransferase activity. As PRMT1 is the primary enzyme catalyzing asymmetric dimethylation of H4R3me2a, we found that FOSL1 regulates PRMT1-mediated H4R3me2a deposition and specifically modulates the ADMA modification of PABPN1. Furthermore, we established that FOSL1 positively regulates global m⁶A methyltransferase activity in a PRMT1-dependent manner, influencing the expression and function of key RNA methyltransferase METTL3 and its target transcripts involved in DDR and tumor progression. These findings collectively establish CAPS as a novel downstream effector in the PRMT1-mediated radiation response, revealing a multi-layered epigenetic and post-transcriptional regulatory network underpinning GBM radioresistance. Complementing our findings, existing literature indicates that CAPS knockdown attenuates MYPT1 phosphorylation at Ser507, leading to reduced PLK1 activity-a key regulator of G2/M transition [33]. These earlier findings help elucidate the mechanism underlying CAPS-mediated cell cycle regulation, suggesting that targeting CAPS may induce cell cycle arrest and provide therapeutic benefits against GBM.

While this study delineates a novel signaling axis in GBM radioresistance, several limitations should be acknowledged. First, the precise structural basis governing the FOSL1-PRMT1 complex formation remains unresolved, and the detailed mechanisms through which CAPS participates in DDR pathways require further elucidation. Second, although we demonstrated that FOSL1 and PRMT1 modulate METTL3 expression and global m⁶A activity, the exact functional consequences of these changes on specific downstream targets, such as PARP1, SIRT1, TSC1, IRF4, and SNAP29, in the context of radiation response need more direct validation. Third, our findings, primarily derived from cell line models and biochemical assays, necessitate further in vivo confirmation using patient-derived xenograft models to better recapitulate tumor heterogeneity and microenvironmental influences. Finally, the therapeutic potential of targeting this axis requires comprehensive preclinical evaluation, including systematic assessment of normal tissue toxicity and potential off-target effects, before clinical translation can be considered. Importantly, while earlier reports have established FOSL1's involvement in various malignancies including cholangiocarcinoma [43] and head and neck squamous cell carcinoma [44], and documented correlations between PRMT1 expression and unfavorable GBM outcomes [45–48], the conceptual advances presented here still await rigorous translational validation. These include the possible repurposing of established FOSL1-targeting agents, such as LY‑1816, which was originally developed for pancreatic cancer [49], for GBM treatment. Additionally, the expression profiles of these signaling components may offer utility as predictive biomarkers for radiation sensitivity, aligning with PRMT1's documented prognostic significance across multiple malignancies [35].

Promising investigative avenues emerging from this work include the structural characterization of the FOSL1-PRMT1 interface to enable rational inhibitor design, comprehensive mapping of CAPS interactors within DNA repair complexes, and functional validation of m⁶A-mediated regulation in patient-derived models. Furthermore, exploring the crosstalk between this axis and other resistance-related pathways—such as immune evasion or metabolic reprogramming—could provide a more integrated view of GBM adaptation to therapy. Continued examination of the molecular architecture and clinical translation potential of the FOSL1-PRMT1-CAPS pathway represents a crucial next step toward developing novel combination strategies to overcome radioresistance in this aggressive malignancy.

In summary, this study establishes the FOSL1-PRMT1-CAPS axis as a central coordinator of radiotherapy resistance in GBM, integrating epigenetic regulation, RNA methylation, and DNA repair. We provide mechanistic evidence that FOSL1, through direct interaction with PRMT1, enhances histone H4 and PABPN1 methylation, upregulates global m⁶A methyltransferase activity, and subsequently activates both HR and NHEJ pathways. These events collectively promote DNA repair efficiency and invasive potential in irradiated GBM cells. Our work not only expands the current understanding of therapeutic resistance mechanisms but also highlights the translational relevance of this pathway. The expression profiles of FOSL1, PRMT1, and CAPS may serve as predictive biomarkers for radiotherapy response, while the multi-nodal architecture of the axis offers several therapeutic access points, including the repurposing of existing FOSL1-targeting agents and the development of selective PRMT1 inhibitors.

## Materials and methods

### Cell culture and irradiation

We purchased the GBM cell lines U251 and LN229 from the Shanghai Cell Bank of the Chinese Academy of Sciences and authenticated them using the STR profiling method. Stable radiation-tolerant U251R and LN229R cell lines were developed from U251 and LN229 parental cell lines after 30 daily 2 Gy X-ray irradiation (usually five times per week) at a dose rate of 1.9 Gy per min using an EX-23 irradiator (Varian Medical Systems) until the total dose reached 60 Gy. All cellular lines were maintained in DMEM supplemented with 10% fetal bovine serum (FBS, Gibco, USA). The cells were maintained at 37 °C in a humidified atmosphere with 5% CO_2_. Routine checks were conducted for the presence of mycoplasma contamination in the cells, and all outcomes consistently confirmed the absence of infection.

### Clinical specimens

We collected 7 tumor specimens patients diagnosed with GBM in The Second People’s Hospital of Wuxi in China from January 2022 to December 2024. These patients had not undergone any anti-tumor treatments such as chemotherapy or radiotherapy prior to the specimen collection. In addition, 6 tumor specimens and corresponding biopsy specimens were collected from patients with GBM who underwent surgical resection following radiotherapy in the Second People’s Hospital of Wuxi in 2020. Informed consents were obtained from all patients or their family members. Human studies were performed in accordance with the Declaration of Helsinki, and the use of these human samples and data for research purposes was approved by the Ethics Committee of the Hospital.

### RT-qPCR

Total RNA was extracted using TRIzol reagent (Cat# R711-01, Vazyme, Nanjing, China) following the manufacturer's instructions. Single-strand complementary DNA (cDNA) was generated using a reverse transcription kit (HiScript III All-in-one RT SuperMix Perfect for qPCR, Cat# R333-01, Vazyme, Nanjing, China). RT-qPCR was then performed with ChamQ Universal SYBR qPCR Master Mix (Cat# Q711-02, Vazyme, Nanjing, China) on a Bio-Rad CFX96 system using 96-well plates. The sequences of primers used in this study are listed in Supplementary Table S2.

### Western blot analysis

Cellular protein samples were lysed using a cell lysis buffer (Cat# P0013B, Beyotime, Shanghai, China) containing 1 mM of phenylmethanesulfonyl fluoride (PMSF; Cat# ST507, Beyotime, Shanghai, China) along with protease and phosphate inhibitors (Cat# A32961, Thermo Fisher Scientific, Shanghai, China). Equal volumes of protein samples were separated using 12% SDS-PAGE gels and transferred to PVDF membranes (Millipore, MA, USA). After blocking with 5% non-fat milk (Cat# P0216, Beyotime, Shanghai, China), the samples were incubated with the primary antibody overnight, followed by binding with rabbit peroxidase-conjugated secondary antibody the next day. Supplementary Table S3 provides a comprehensive listing of the pertinent details concerning the antibodies used.

### Plasmid construction and transfection

The PRMT1 and CAPS overexpression plasmid constructed by the use of pcDNA3.1 were purchased from GenePharma (Shanghai, China). Lipofectamine 3000 (Invitrogen, Life Technologies, USA) was used as the transfection reagent for all cell transfection operations when the fusion degree was 70%−90%. The NC plasmids were used as control vectors.

### Establishment of stable cell lines

Lentiviruses harboring FOSL1, PRMT1, or CAPS, and those encoding shRNA targeting these genes, were acquired from Genomeditech. Based on the detailed how-to guidelines provided by the manufacturer, we employed lentiviral vectors for efficient cell transfection. To establish stable cell lines, cells were selected using 2 µg/ml of puromycin (Solarbio, Beijing, China) based on their resistance to this antibiotic. The identities of the selected cell lines were further verified through western blotting. The specific shRNA sequences used are elaborated and described in Supplementary Table S1.

### Cell viability assay

After resuspension, the cells were evenly seeded into 96-well dishes with a cell density range of 1000–3000 cells per well, followed by a 24-h incubation period. Afterward, they were subjected to a range of X-ray doses of 0, 2, 4, and 8 Gy. Subsequently, 10 μL of Cell Counting Kit-8 (CCK-8) reagent (NCM, Suzhou, China) was added to each well, followed by incubation for 1 h to assess cell viability. Next, the absorbance values were measured at a wavelength of 450 nm using a microplate reader (Thermo Fisher Scientific, Shanghai, China). The half maximal inhibitory concentration (IC_50_) was determined using GraphPad Prism 9 software.

### Co-IP and silver staining

The cells were initially washed with PBS and then lysed using RIPA lysis buffer containing protease and phosphatase inhibitors (Cat# A32961, Thermo Fisher Scientific, Shanghai, China). The cell lysates were subjected to a pre-clarification step using Protein A/G-agarose beads (Cat# SC-2003, Santa Cruz Biotechnology, USA). Subsequently, 1 mL of the supernatant was incubated with 1 µg of either anti-FOSL1/-PRMT1 antibody or anti-IgG antibody (Cat# 3423, Cell Signaling Technology, USA, diluted 1:100) for a duration of 16 h. Following incubation, 40 μL of Protein A/G agarose beads (Cat# SC-2003, Santa Cruz Biotechnology, USA) were added to the mixture and incubated at 4 °C overnight. Subsequently, the sample was centrifuged and washed four times with pre-chilled PBS. Next, the sample was heated at 100 °C for 10 min before loading it onto an SDS-PAGE gel. The immunoprecipitated complexes were electrophoresed and analyzed on a 12% SDS-PAGE gel, before analyzing via immunoblotting. Silver staining of the gels was performed following the manufacturer’s instructions using the PAGE Gel Silver Stain Reagent Set of Solarbio (Cat# G7210, Beijing, China).

### Comet assay

The Comet assay procedure was conducted using reagents sourced from a specific Comet Assay kit (Cat# KGA1302-100, KeyGen BioTECH, Jiangsu, China) following the manufacturer’s guidelines; the cells were briefly combined with agarose matrices. Each possessed distinct melting points in an appropriate ratio and was subsequently spread onto the slides designed for comet assays. After immersion in a sequence of buffers, the slides were subjected to electrophoresis at a voltage of 25 V for 30 min. The slides were stained with a red PI solution. Quantitative analysis was conducted using CASP Software, and at least 20 cells were evaluated in each experimental cohort.

### Immunofluorescence staining

Cells were seeded onto glass slides (Cat# BS-14-RC, Biosharp, Anhui, China) and positioned within 24-well plates. The mice were immersed in 4% paraformaldehyde for fixation for approximately 10 min, permeabilized using 0.5% Triton X-100 for a duration of 20 min, and blocked using 10% goat serum for 1 h. The cells were incubated overnight at 4 °C with a primary antibody. This was followed by the application of a fluorescent secondary antibody and an antifluorescence reagent incorporating DAPI (Solarbio, Beijing, China) for staining. Finally, images were acquired using an Olympus FV3000 confocal microscope (Japan).

### Immunohistochemistry (IHC)

All tissue specimens were fixed with formalin or 4% paraformaldehyde, immersed in paraffin wax, and sectioned. The sections were immersed in xylene for dewaxing and hydration with 100%, 95%, and 80% alcohol. The sections were incubated with 10% goat serum albumin for a duration of 1 h, after which the relative primary antibodies were added and the sections were incubated overnight. Subsequently, the tissue sections were incubated with the secondary antibody for 2 h. Following incubation, the sections were washed with pure water, stained with DAB, and then observed under a microscope. After washing with water, hematoxylin staining was performed, followed by graded alcohol dehydration and neutral resin sealing.

### Total RNA sequencing

Total RNA was extracted using Trizol Reagent (Invitrogen Life Technologies), and its concentration, quality, and integrity were assessed using a NanoDrop spectrophotometer (Thermo Scientific). Sequencing libraries were constructed from 3 µg of total RNA per sample according to the following procedure. Briefly, mRNA was enriched from total RNA using poly-T oligo-attached magnetic beads. The purified mRNA was then fragmented in an Illumina proprietary fragmentation buffer with divalent cations under elevated temperature. First-strand cDNA was synthesized with random hexamers and SuperScript II reverse transcriptase, followed by second-strand cDNA synthesis with DNA Polymerase I and RNase H. The resulting cDNA fragments were end-repaired, adenylated at their 3' ends, and ligated to Illumina PE adapter oligonucleotides. Library fragments of 400–500 bp were size-selected using the AMPure XP system (Beckman Coulter) and then enriched by 15 cycles of PCR with Illumina PCR Primer Cocktail. The final library quality and quantity were evaluated using the Agilent High Sensitivity DNA kit on a Bioanalyzer 2100 system (Agilent Technologies). Sequencing was performed on an Illumina NovaSeq 6000 platform at Shanghai Personal Biotechnology Co. Ltd.

### Immunoprecipitation-mass spectrometry (IP-MS)

Proteins were extracted and cells were lysed using SDT buffer (4% SDS, 100 mM Tris–HCl, 1 mM DTT, pH 7.6). The total protein concentration was determined with a BCA Protein Assay Kit (Bio-Rad, USA). Protein aliquots were prepared and stored at −80 °C until further analysis. For electrophoresis, 20 µg of protein per sample was mixed with 5 × loading buffer and denatured at 100 °C for 5 min. The samples were separated on 12.5% SDS–polyacrylamide gels at a constant current of 14 mA for 90 min. Proteins were visualized by Coomassie Blue R-250 staining, followed by in-gel digestion with trypsin. The resulting peptides were desalted using C18 solid-phase extraction cartridges (Empore™ SPE Cartridges C18, standard density, 7 mm inner diameter, 3 mL bed volume; Sigma), dried by vacuum centrifugation, and reconstituted in 40 µL of 0.1% (v/v) formic acid. Liquid chromatography-tandem mass spectrometry (LC–MS/MS) analysis was performed on a timsTOF Pro instrument (Bruker) coupled online to a Nanoelute nanoflow HPLC system (Bruker Daltonics) with a 45-min analytical gradient. Peptides were loaded onto a homemade reversed-phase C18 analytical column (25 cm × 75 μm i.d., 1.9 μm particles) equilibrated with solvent A (0.1% formic acid in water) and eluted with a linear gradient of solvent B (0.1% formic acid in acetonitrile) at a constant flow rate of 300 nL/min. Mass spectrometry data were acquired in positive ion mode using parallel accumulation-serial fragmentation (PASEF). Full-scan MS spectra were collected over an m/z range of 100–1700 and an ion mobility (1/k0) range of 0.75–1.35 V·s·cm⁻^2^. Up to 10 PASEF MS/MS scans were acquired per cycle, with a target intensity of 1,500 and a threshold of 2,500. Active exclusion was set to 0.4 min.

### Detection of total m⁶A RNA methyltransferase activity

The total m⁶A methyltransferase activity was quantified using a commercial colorimetric assay kit following the manufacturer's instructions (EpiGentek, USA). Briefly, strip wells were pre-coated with a proprietary nucleic acid substrate containing methyl-accepting adenosine residues. Following the enzymatic reaction, newly formed m⁶A modifications were detected with a high-specificity anti-m⁶A primary antibody. The resulting immunocomplex signal was then amplified with an enhancement solution. Methylation levels, which are directly proportional to the enzymatic activity, were quantified using an ELISA-like colorimetric detection method. Absorbance was measured at 450 nm with a multi-mode microplate reader (BIO-TEK, USA), with all readings completed within 10 min after terminating the reaction.

### RNA methylation co-immunoprecipitation (MeRIP)

The MeRIP Kit (BersinBio, China) was used to carry out the experiment according to the manufacturer’s instructions. In brief, total RNA was isolated from approximately 2 × 10⁷ glioma cells and subjected to fragmentation. The resulting RNA fragments were allocated into three experimental groups: Input, IP, and IgG. The IP group received 5 µg of anti-m⁶A antibody, while the IgG control group was treated with an equivalent amount of anti-IgG antibody. Both mixtures were incubated with constant rotation at 4 °C for 2–4 h. Pre-washed protein A/G magnetic beads were then combined with the antibody-RNA complexes from the IP and IgG groups. Following the immunoprecipitation step, the samples were treated with proteinase K. The beads were subsequently separated, and the supernatant was carefully transferred to RNase-free collection tubes. The immunoprecipitated RNA was finally purified and analyzed via quantitative reverse transcription PCR to evaluate m⁶A enrichment. List of primers used for MeRIP-qPCR analysis are listed in Supplementary Table S4.

### Xenograft tumor model

Male BALB/c nude mice, aged between 4 and 6 weeks, were enrolled and administered a dose of 5 × 10^6^ cells via injection. To induce tumors, we employed four groups of mice, each consisting of six animals, which were injected with distinct cell lines: control cells (LN229R + sh-NC + Vector), FOSL1-knockdown LN229R cells (LN229R + sh1-FOSL1 + Vector), LN229R cells that exhibit both a downregulation of FOSL1 and an upregulation of PRMT1 (LN229R + sh1-FOSL1 + PRMT1), and LN229R cells featuring knockdown of both FOSL1 and CAPS, along with overexpression of PRMT1 (LN229R + sh1-FOSL1 + PRMT1 + sh3-CAPS). After 14 days, MRI (Siemens) was performed on mice to observe the size of the tumor. The length (L) and width (W) of the maximum cross-section of the tumor were recorded, as were the number of imaging layers (H) (0.7 mm per layer). The tumor volume was calculated using the formula: volume (mm^3^) = 4/3 × π × L × W × H. The animal experiments were approved by the Ethics Review Board for Animal Experimentation at the Second People’s Hospital of Wuxi, which is affiliated with the Clinical College of Nantong University in Wuxi.

### KEGG and GO enrichment analysis

Gene Ontology (GO) enrichment analysis was performed using the topGO package, employing the hypergeometric test to calculate p-values. Terms with a p-value < 0.05 were considered significantly enriched, allowing identification of the primary biological functions associated with the differentially expressed genes. Kyoto Encyclopedia of Genes and Genomes (KEGG) pathway enrichment analysis was conducted using the clusterProfiler software (version 3.16.1), with a focus on pathways showing significant enrichment (p-value < 0.05).

### Quantitative and statistical analysis

All statistical analyses were performed using GraphPad Prism (version 9.0). For comparisons between two groups, unpaired or paired Student's t-tests were applied as appropriate and indicated in the figure legends. Survival analysis was conducted using the Kaplan–Meier method, and differences between survival curves were assessed with the log-rank test. In all analyses, a p-value of less than 0.05 was considered statistically significant. Quantitative data are presented as the mean ± standard error of the mean (SEM).

## Supplementary Information

Additional file 1. Supplemental Information includes extended materials and methods and supplementary figures (Fig. S1-S12).

Additional file 2.

Additional file 3.

## Data Availability

All data included in this study are available from the corresponding author upon reasonable request (zhaoxudong623@njmu.edu.cn).
