# Supplementary material for: FOSL1-PRMT1 transcriptional-epigenetic circuit promotes glioblastoma radioresistance via calcyphosine-mediated DNA repair and invasion
Source: Mol Biomed. 2025 Dec 22;6:147. doi: 10.1186/s43556-025-00394-0 (PMC12719381; doi:10.1186/s43556-025-00394-0)
Supplement: Supplementary file 1 — Additional file 1. Supplemental Information includes extended materials and methods and supplementary figures (Fig. S1-S12). [file 43556_2025_394_MOESM1_ESM.docx]

**Supplementary document**

**Title page**

**FOSL1-PRMT1 Transcriptional-Epigenetic Circuit Promotes Glioblastoma Radioresistance via Calcyphosine-Mediated DNA Repair and Invasion**

**Authors:** Yating Zhang ^1, 2, #^, Jiajia Tian ^1, 2, #^, Shuai Wu ^3, #^, Yiting Zhou ^4^, Zhongyuan Bao ^5, 6^, Yi Zhu ^3^, Peng Wang ^3^, Zixiang Liu ^5, 6^, Pengpeng Li ^5, 6^, Zhenxing Tao ^5, 6^, Zengli Miao ^5, 6^, Xiaojie Lu ^1, 2, 5, 6, *^, Xudong Zhao ^3, 5, 6, *^

**Affiliations:**

^1^ *Neuroscience Center, Wuxi School of Medicine, Jiangnan University, Wuxi, Jiangsu Province, 214122, China*

^2^ *Department of Neurosurgery, Jiangnan University Medical Center, Wuxi, Jiangsu Province, 214122, China*

^3^ *Department of Neurosurgery, Medical School of Nantong University, Nantong University, Nantong, 226019, China*

^4^ *Department of Intervention Therapy, The Affiliated Hospital of Jiangnan University, Wuxi, 214002, China*

^5^ *Department of Neurosurgery, Wuxi No. 2 People’s Hospital, Wuxi, 214002, China*

^6^ *Wuxi Neurosurgical Institute, Wuxi, 214002, China*

* Corresponding author.

1. *mail address*: [zhaoxudong623@njmu.edu.cn](mailto:(zhaoxudong623@njmu.edu.cn)) (X. Zhao) and [xiaojieluwuxi@163.com](mailto:xiaojieluwuxi@163.com) (X. Lu)

^#^ These authors contributed equally to this work

**Supplementary Figures**


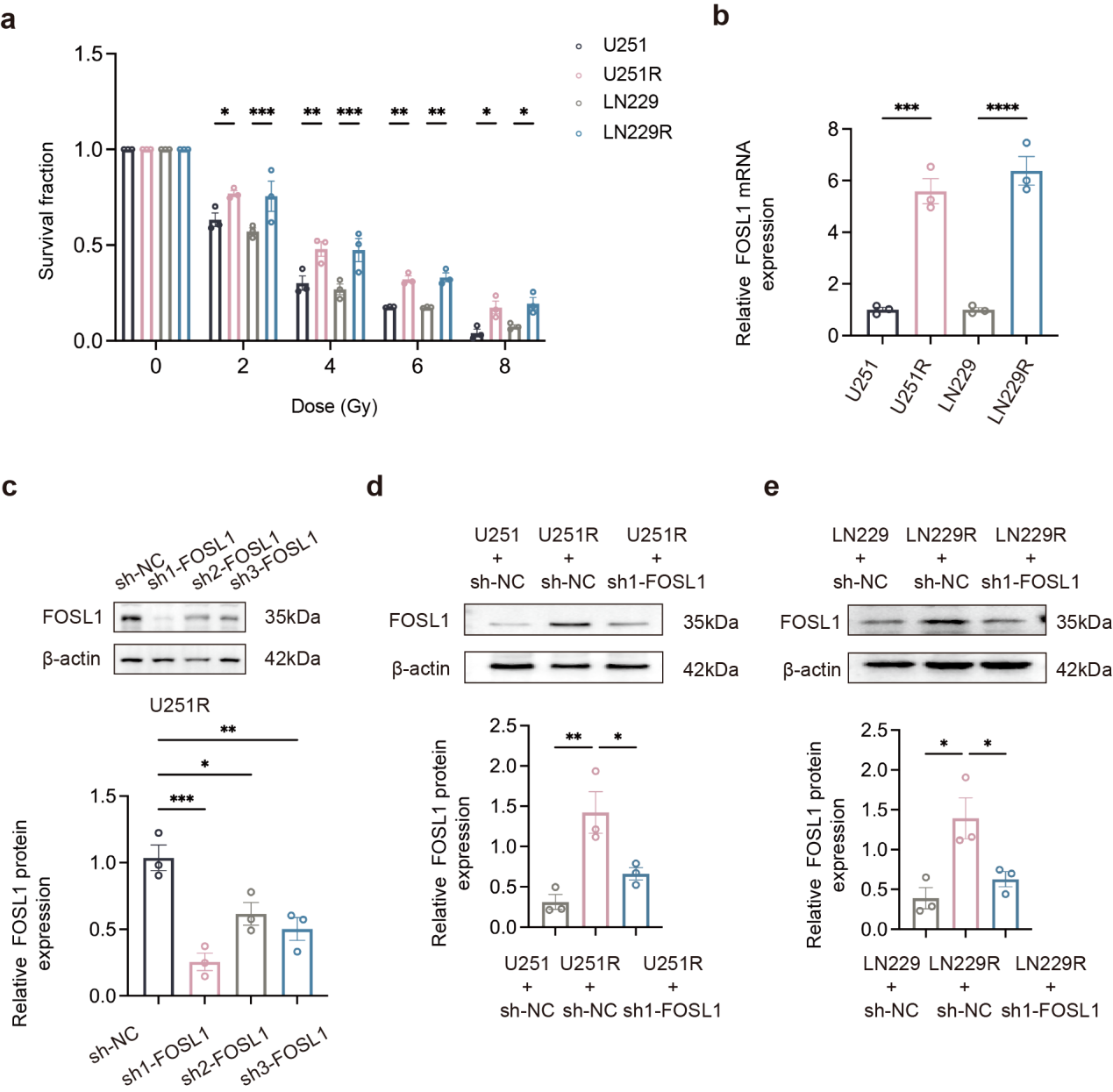


****Fig. S1.** Characterization of radioresistant models and FOSL1 knockdown.** **a** CCK-8 assay measuring radiosensitivity in parental and radioresistant cells. **b** FOSL1 mRNA levels in radioresistant vs. parental cells by RT‑qPCR. **c** FOSL1 protein expression in U251R cells with control or FOSL1‑targeting shRNAs. **d, e** Western blot validation of FOSL1 knockdown efficiency using specific lentiviruses.


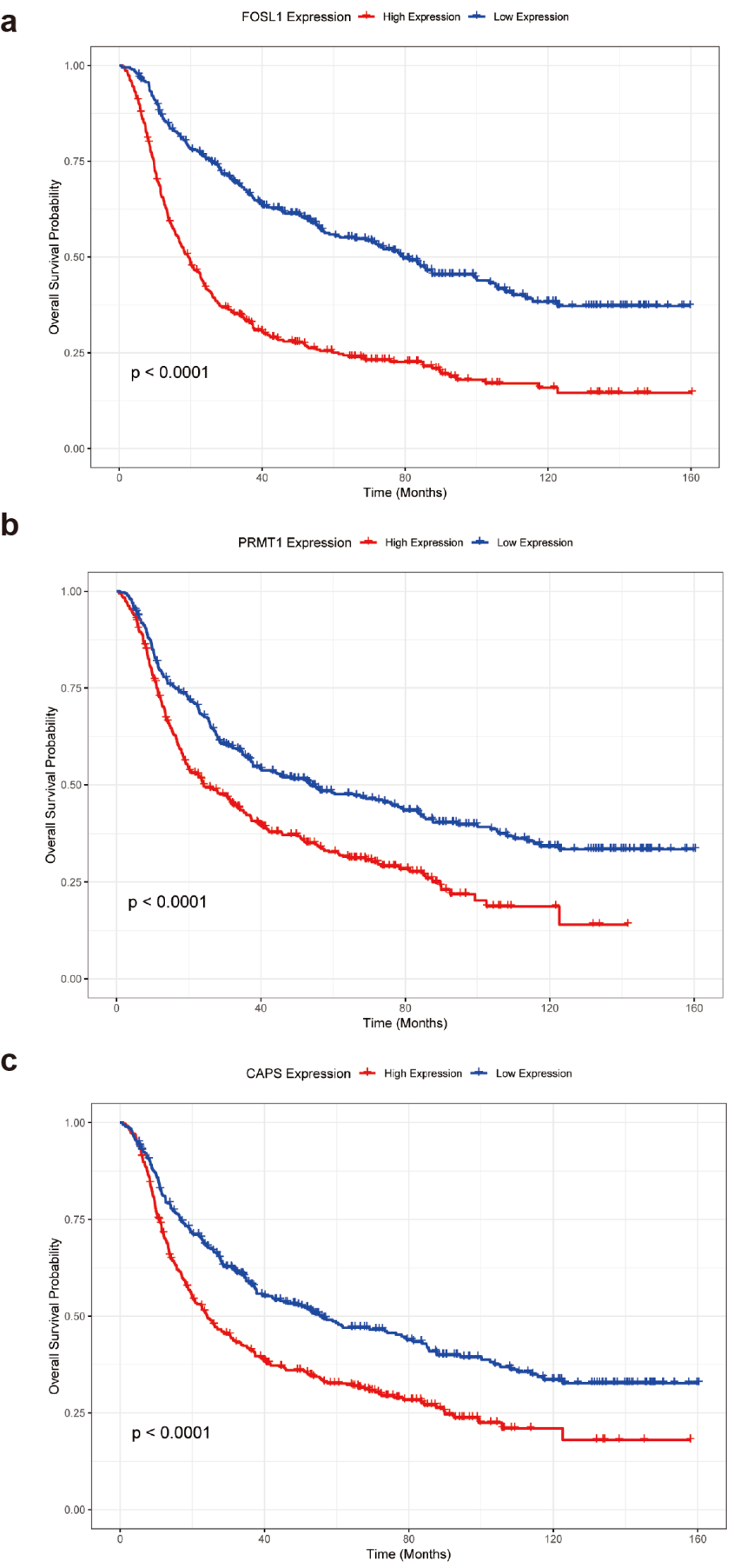


**Fig. S2** Survival analysis of FOSL1, PRMT1, and CAPS in GBM patients.

**a-c** Kaplan-Meier survival curves for GBM patients stratified by FOSL1, PRMT1, CAPS expression levels (data from CGGA).


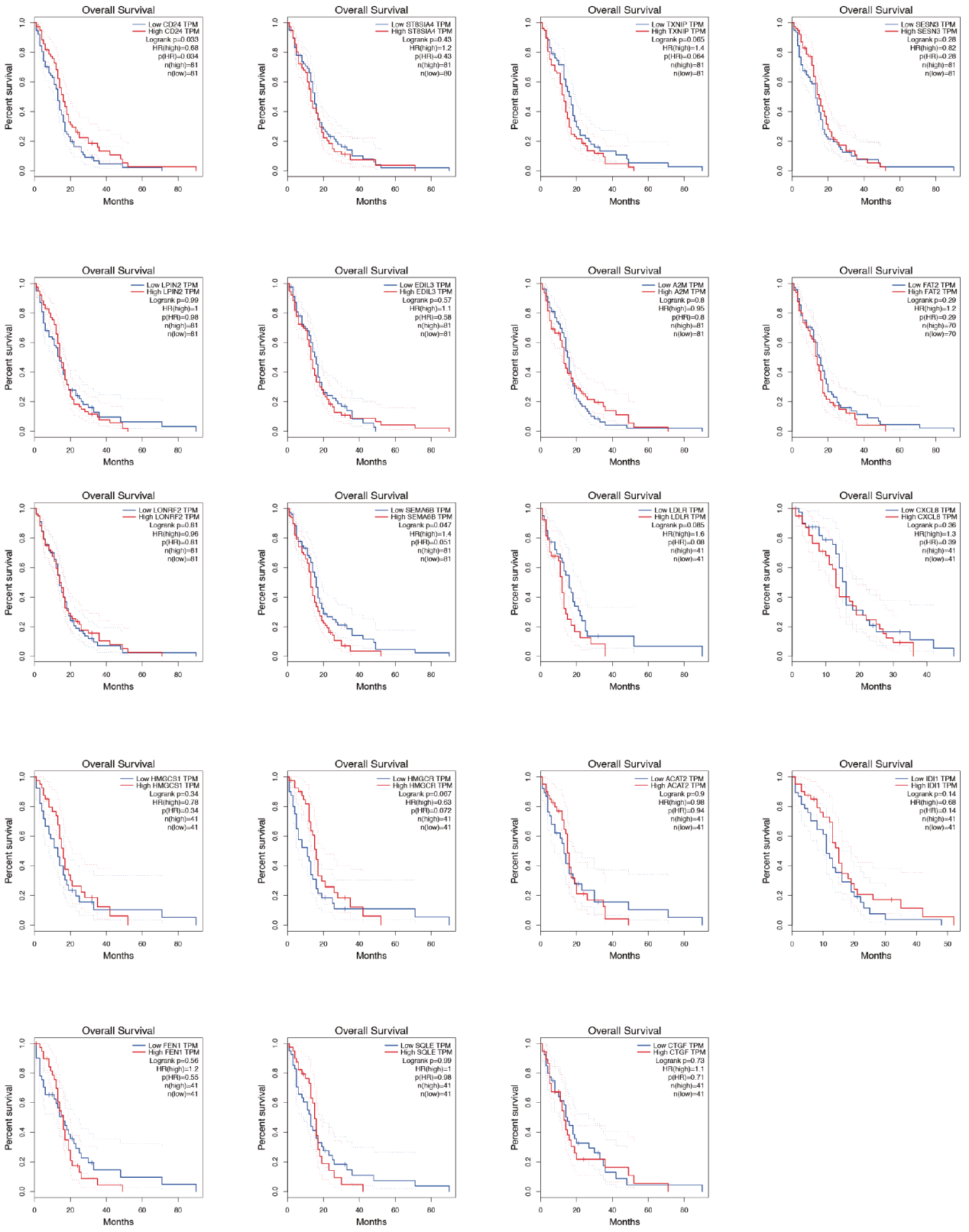


**Fig. S3** The overall survival curve of another 19 genes in the volcano plot of Fig. 1c

**
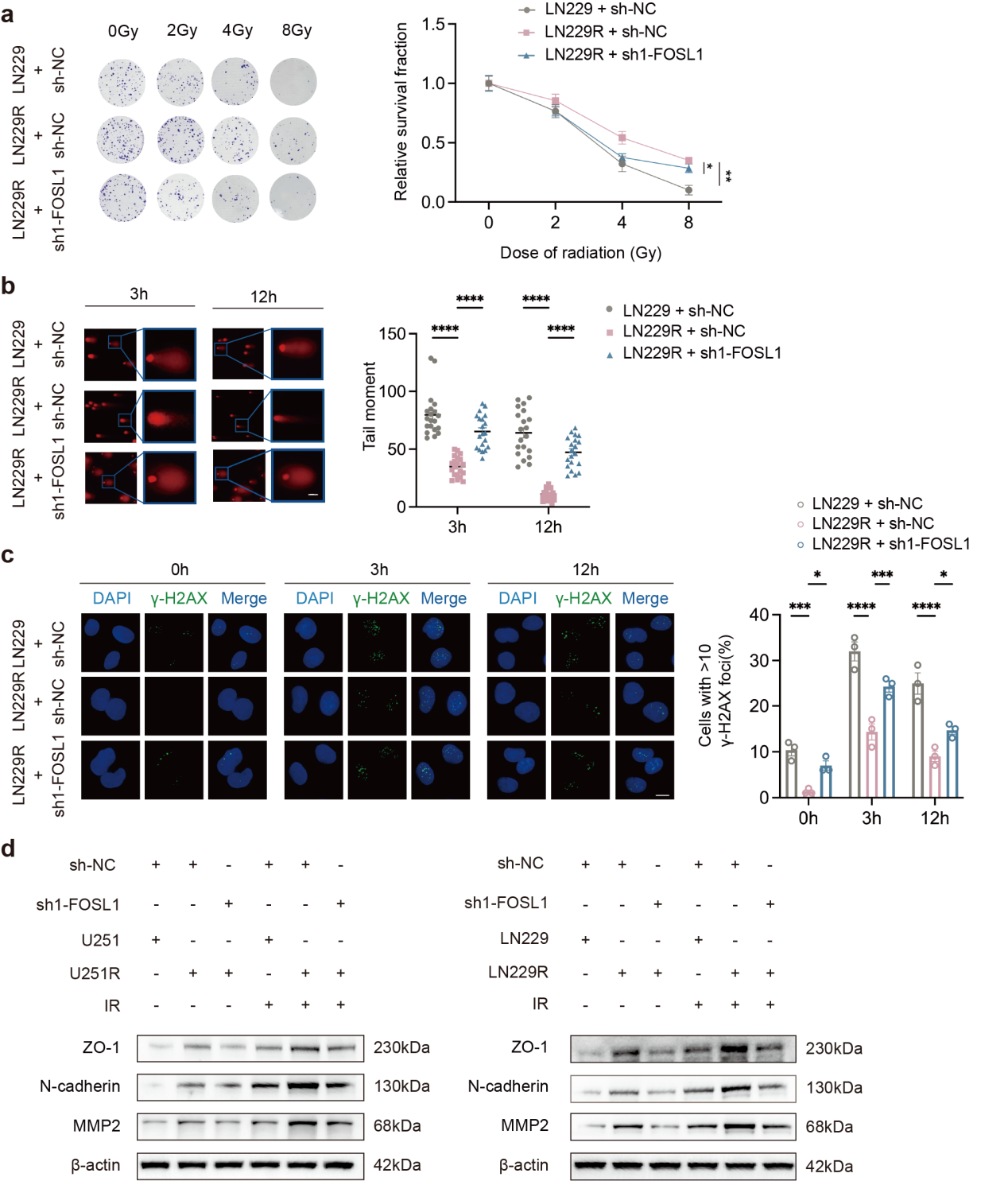
**

**Fig. S4** FOSL1 enhances DNA damage repair to confer radioresistance in glioblastoma. **a** Colony formation assays of control and FOSL1-knockdown cells after exposure to the indicated doses of irradiation. **b** Comet assays and **c** representative immunofluorescence images with quantification of γ-H2AX foci in control and FOSL1-knockdown cells at the indicated times post-IR. Scale bars: 20 μm (**b, c**). **d** Western blot analysis of ZO-1, N-cadherin, and MMP2 protein levels under non-irradiated and 4 Gy irradiated conditions. *p < 0.05, ***p < 0.001, ****p < 0.0001.


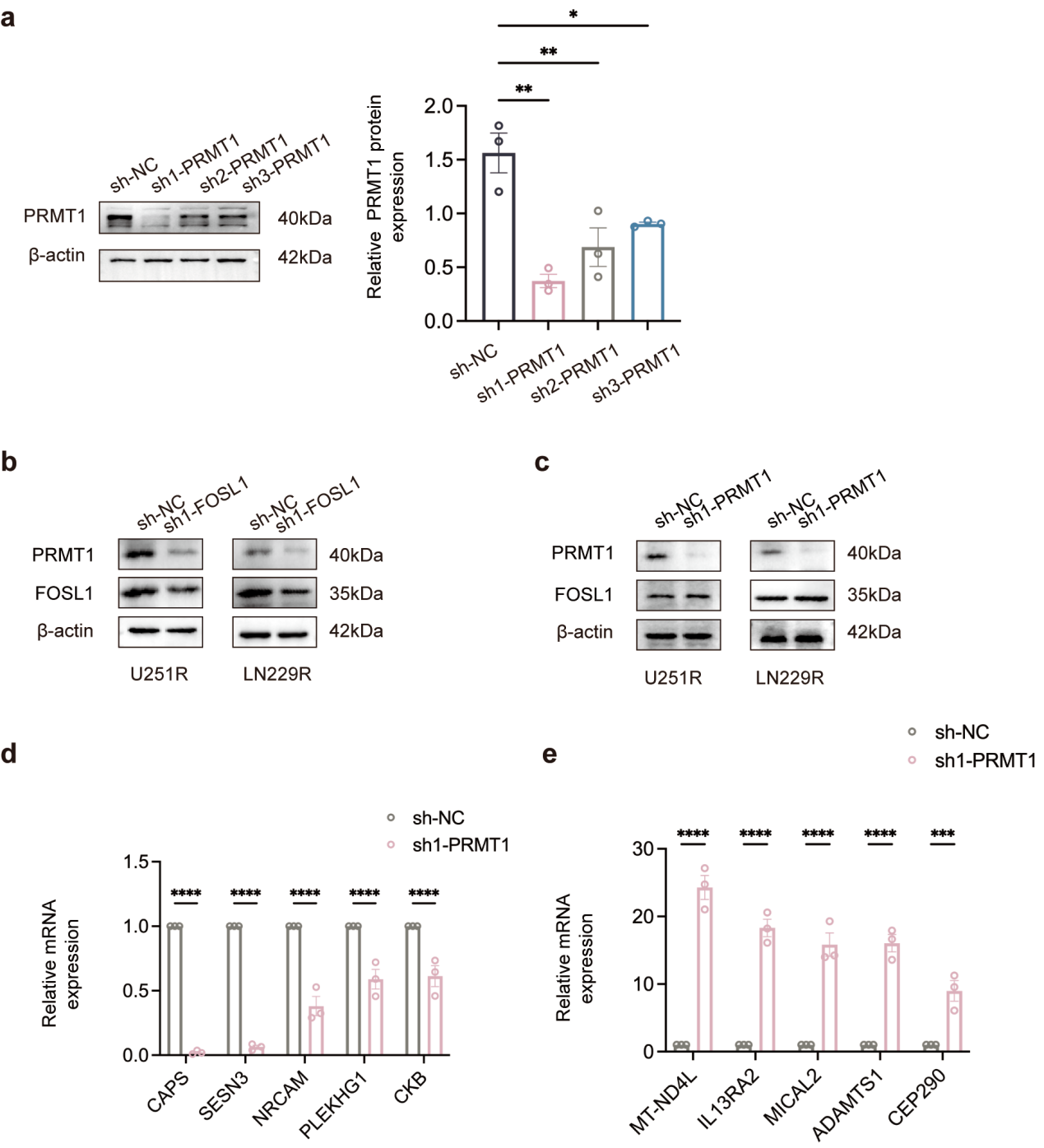


**Fig. S5** Validation of PRMT1 knockdown and screening of downstream targets. **a** Western blot analysis of PRMT1 protein levels in U251R cells stably transduced with control or PRMT1-targeting lentiviruses (sh1/2/3-PRMT1). **b** Western blot analysis of FOSL1 and PRMT1 protein levels in GBM cells stably expressing sh1-FOSL1 or a control vector. **c** Western blot analysis of FOSL1 and PRMT1 protein levels in sh1-PRMT1 GBM cells compared to sh-NC controls. (**d, e**) RT‑qPCR analysis of the top five downregulated and upregulated genes in sh1-PRMT1 U251R cells compared to sh-NC controls. *p < 0.05, **p < 0.01, ***p < 0.001, ****p < 0.0001.


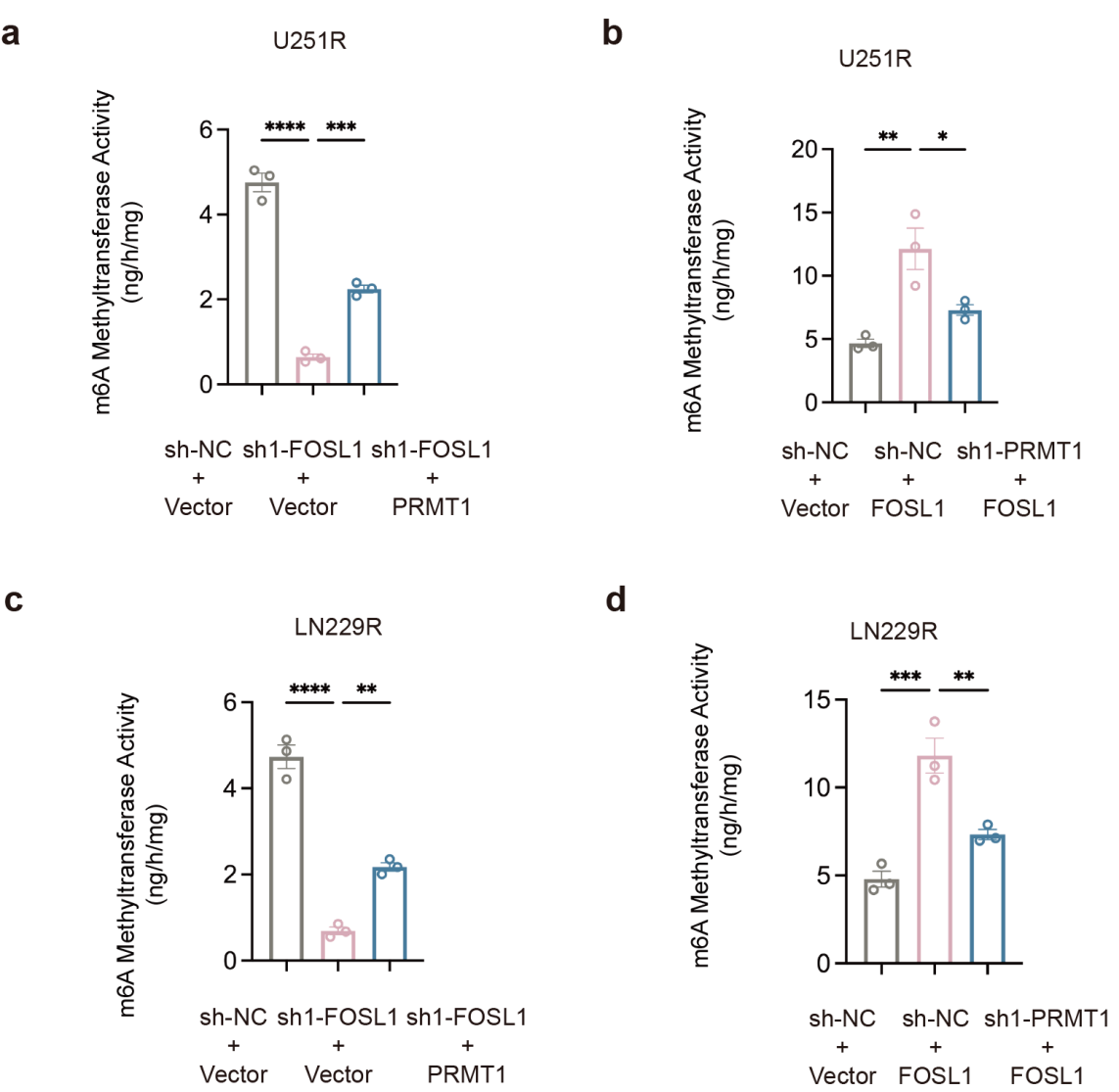


**Fig. S6** The m6A methylation activity and overall m6A methylation levels. **a-d** The m6A methyltransferase activity.


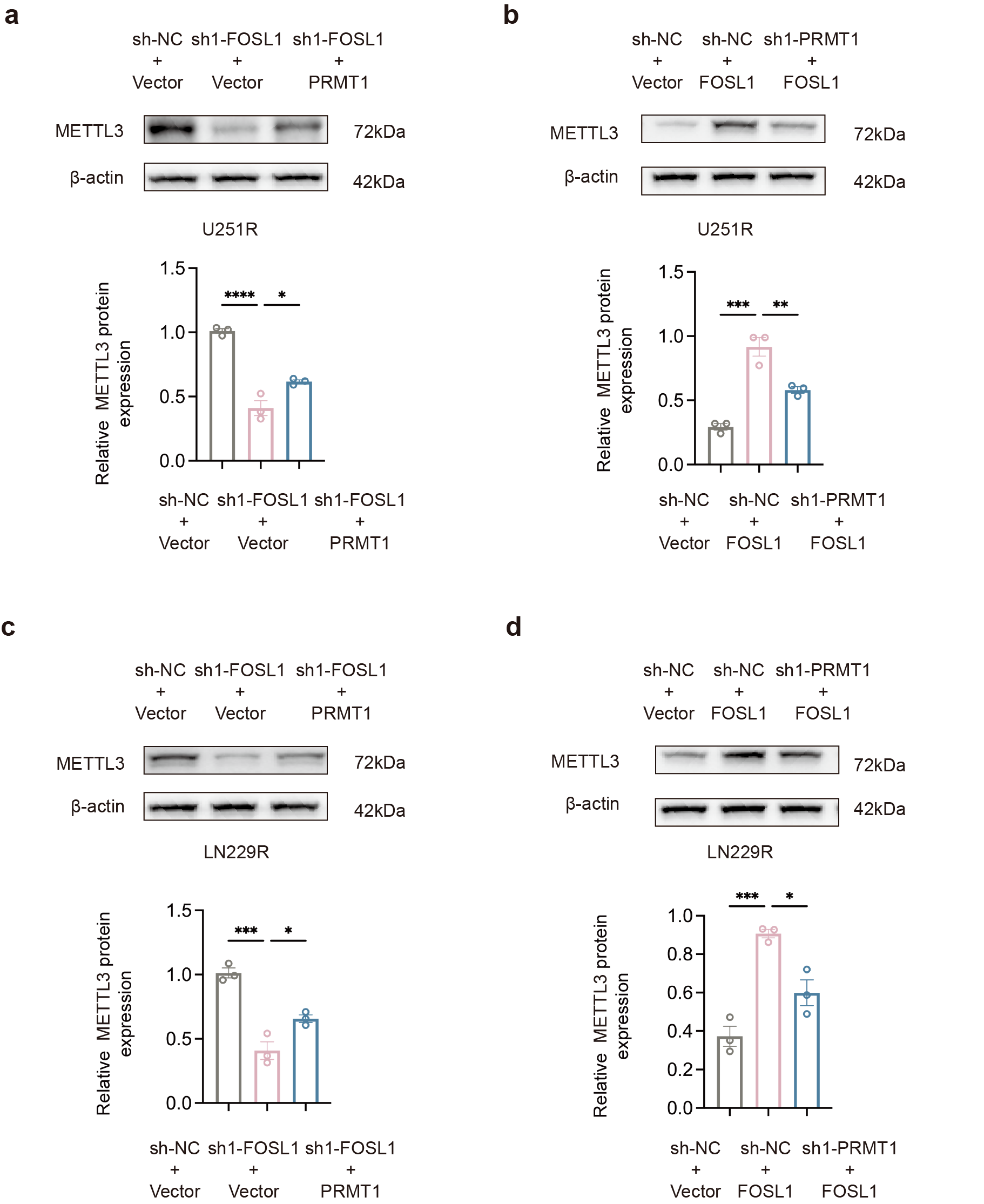


**Fig. S7** METTL3 protein expression is influenced by both FOSL1 and PRMT1 **a-d** Western blot analysis of METTL3 protein levels. *p < 0.05, **p < 0.01, ***p < 0.001, ****p < 0.0001.


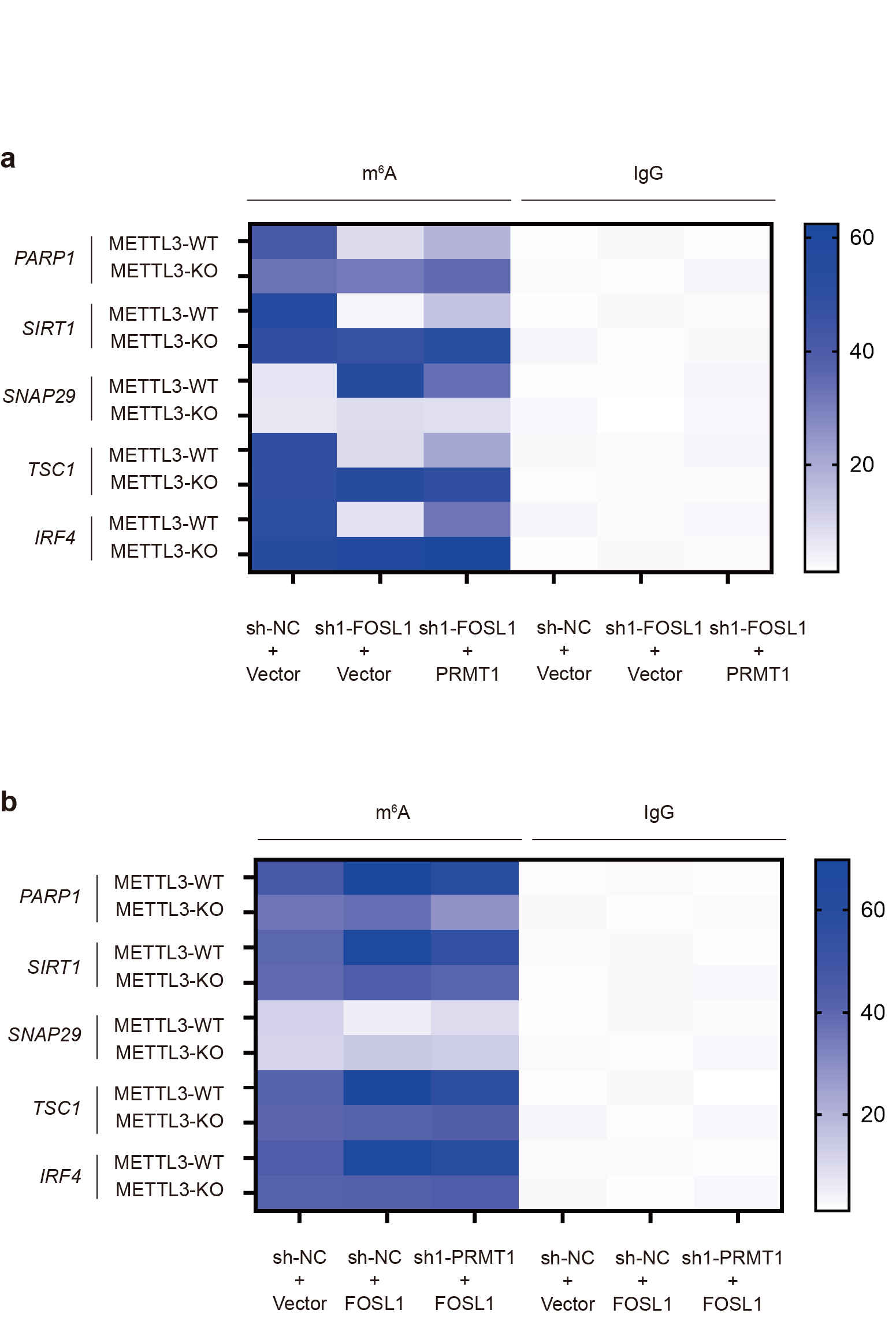


**Fig. S8** The m⁶A enrichment. **a-b** Heatmap of m⁶A enrichment on target mRNAs (MeRIP-qPCR).


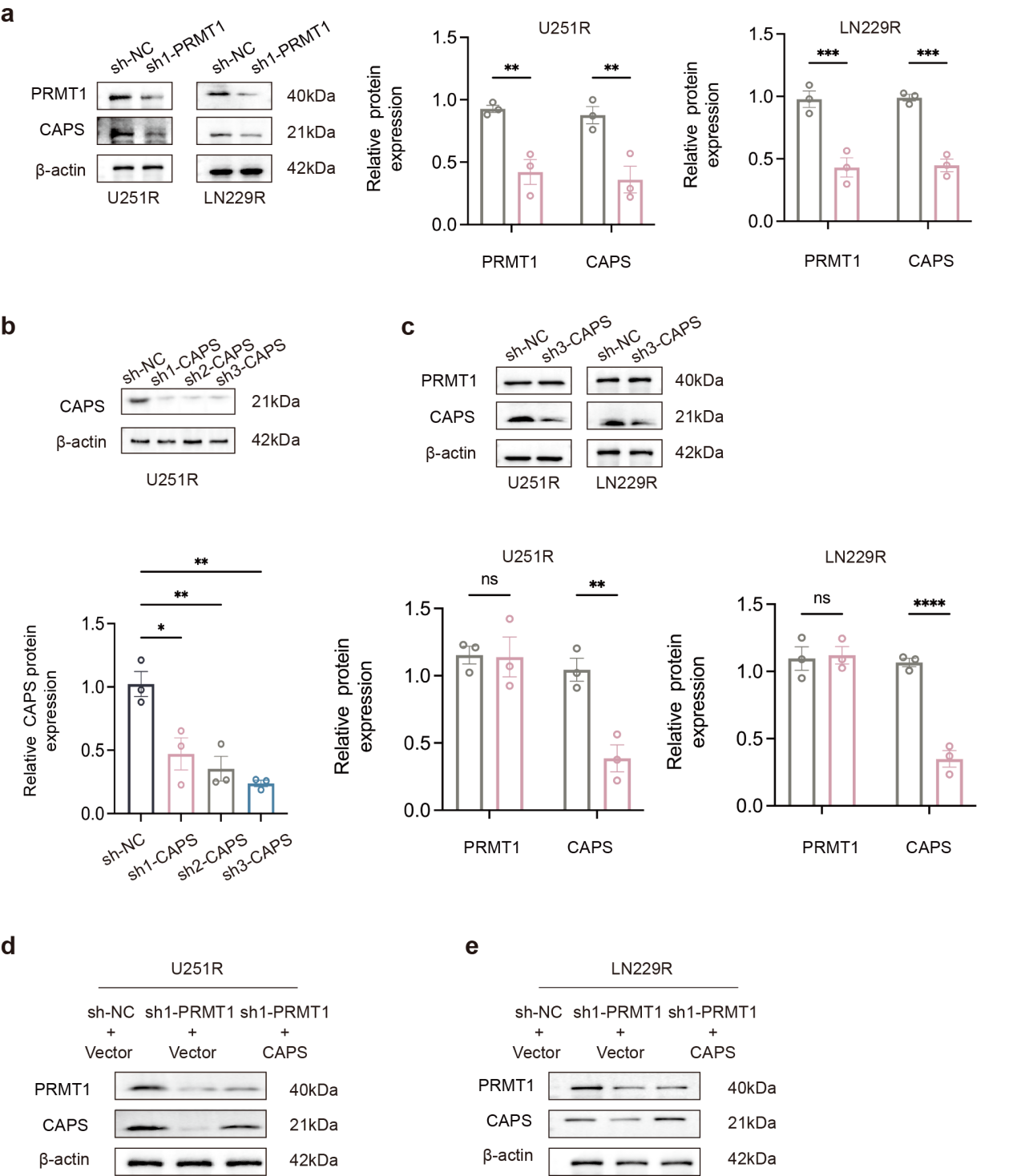


**Fig. S9** Genetic interaction between PRMT1 and CAPS. **a** Effect of PRMT1 knockdown on CAPS protein levels. **b** Validation of CAPS knockdown efficiency. **c** Effect of CAPS knockdown on PRMT1 protein levels. **d, e** Rescue of PRMT1 knockdown phenotype by CAPS overexpression. *p < 0.05, **p < 0.01, ***p < 0.001, ****p < 0.0001.


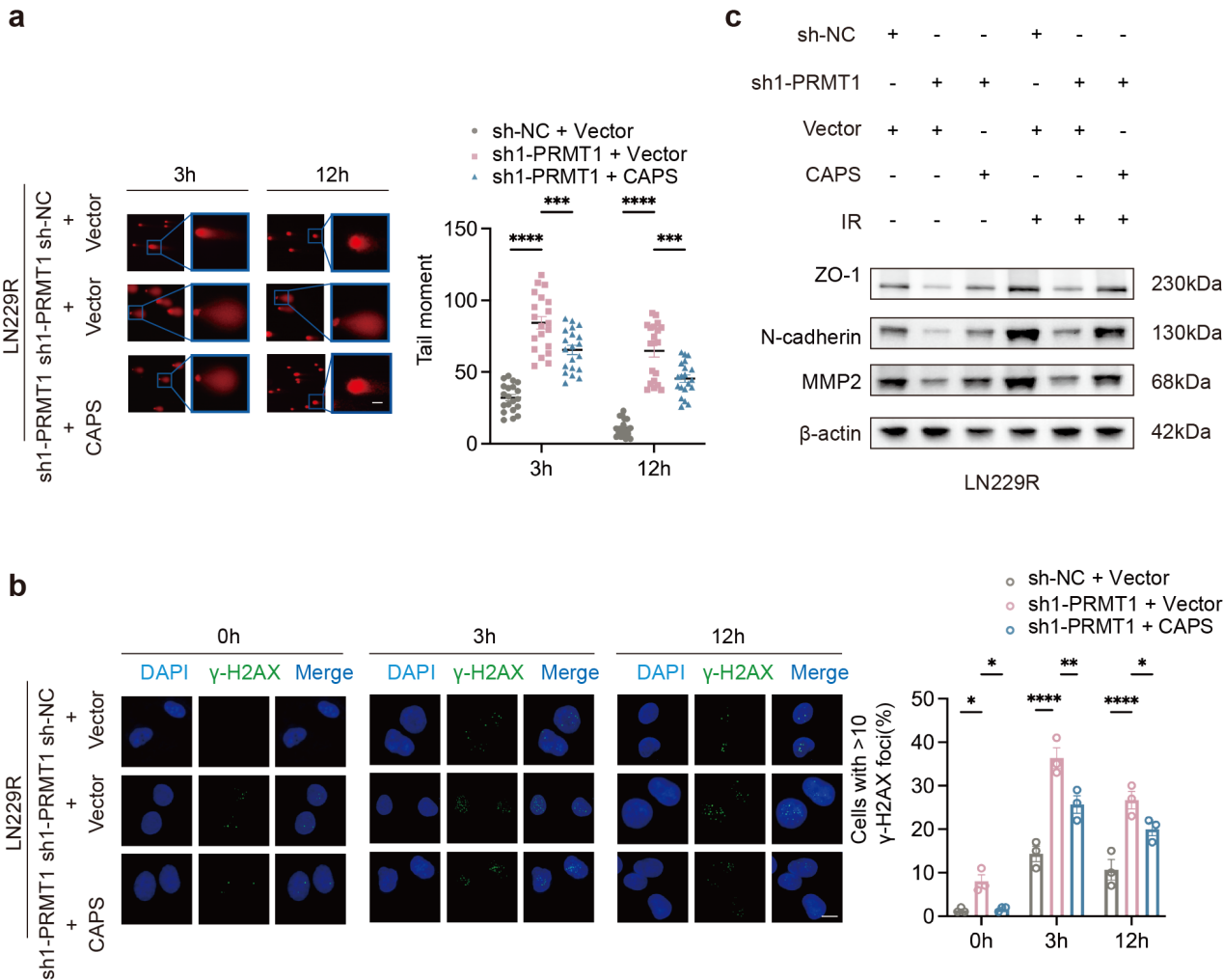


**Fig. 10** PRMT1-CAPS axis confers radioresistance via coordinated DNA repair and invasion. **a** Comet assays with quantification of olive tail moment showing DNA damage in LN229R cells under the indicated conditions post-IR (4 Gy). Scale bar: 20 μm. **b** Immunofluorescence analysis of γ-H2AX foci (green) in LN229R cells under the indicated conditions post-IR. Nuclei (blue, DAPI). Scale bar: 20 μm. **c** Western blot analysis of epithelial-mesenchymal transition (EMT) and invasion markers (ZO-1, N-cadherin, MMP2) in LN229R cells with or without irradiation (4 Gy) under the indicated genetic manipulations. *p<0.05, **p<0.01, ***p<0.001, ****p<0.0001.


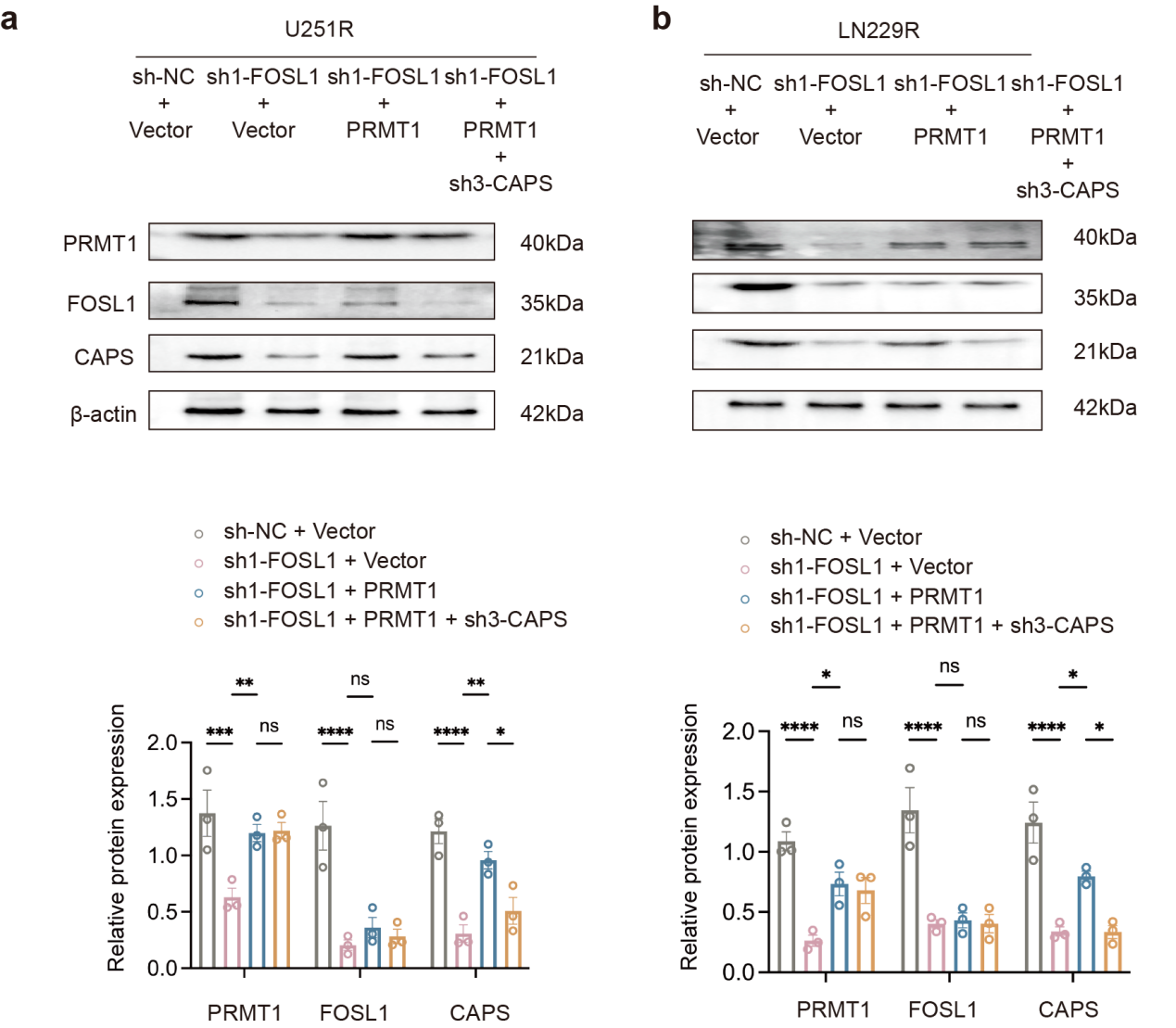


**Fig. S11** CAPS is a functional downstream effector of the FOSL1-PRMT1 axis. **a, b** Western blot analysis validating the protein levels of FOSL1, PRMT1, and CAPS in a series of genetic rescue experiments. *p < 0.05, **p < 0.01, ***p < 0.001, ****p < 0.0001.

**
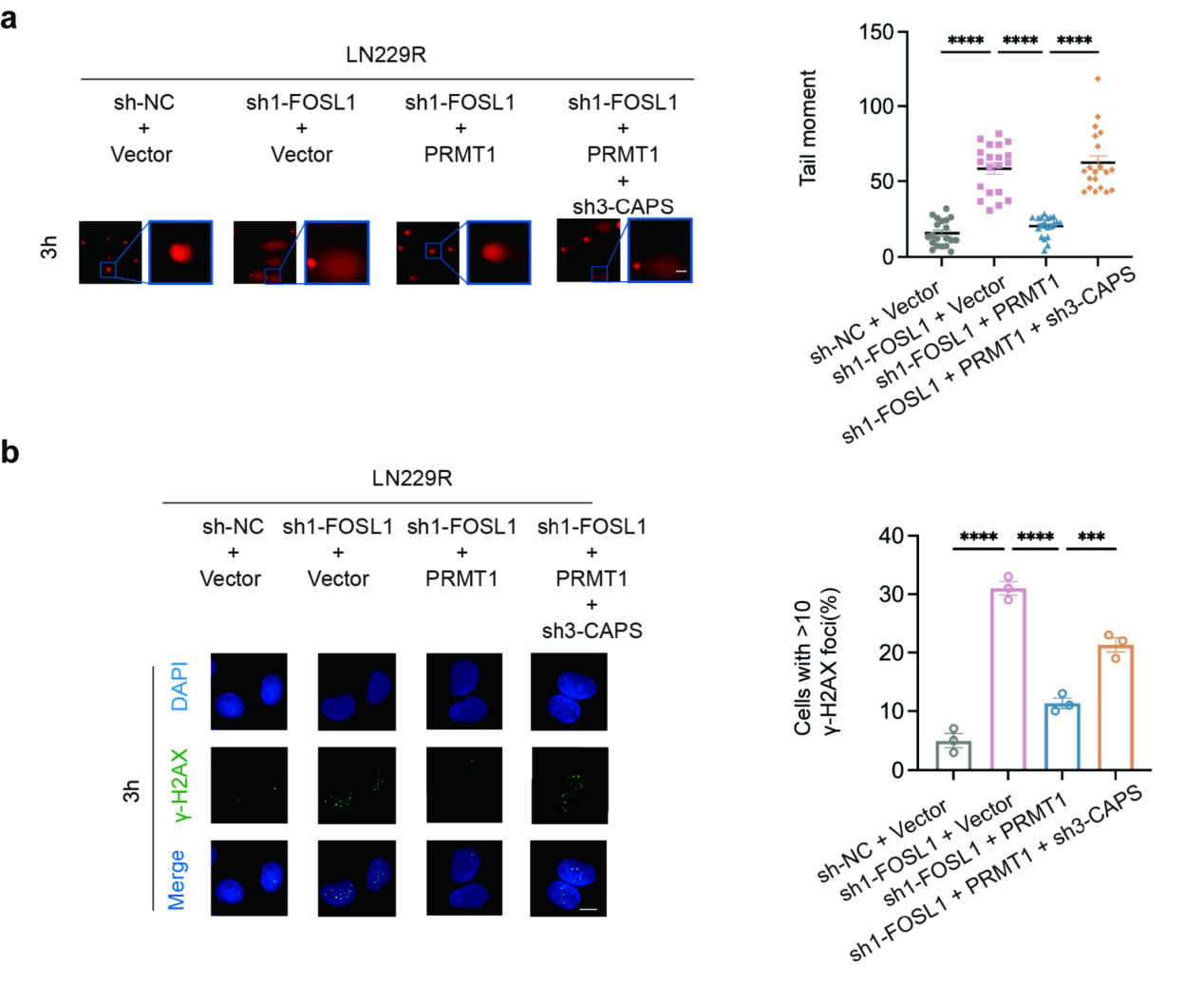
**

**Fig. S12** Functional analysis of the FOSL1-PRMT1-CAPS axis in DNA repair. **a** Comet assays assessing DNA damage in LN229R cells under the indicated treatment conditions after 4 Gy irradiation. Quantification of the olive tail moment is shown in the adjacent panel. Scale bar: 20 μm. **b** Representative immunofluorescence images of γ-H2AX foci (green) in LN229R cells under the indicated treatment conditions after 4 Gy irradiation. Quantification of foci per cell is shown in the adjacent panel. Nuclei were counterstained with DAPI (blue). Scale bar: 20 μm. *p < 0.05, **p < 0.01, ***p < 0.001, ****p < 0.0001.

**Supplementary Tables**

**Supplementary Table S1.** The sequences of lentivirus or plasmids used in this study

| **Gene** | **Sequence (5’ to 3’)** |
| --- | --- |
| **shRNA sequences** |  |
| sh1-FOSL1 | TCCAGCTATGGAAGAAGAAGC |
| sh2-FOSL1 | GGCGCTCATCGATTTCCAGAA |
| sh3-FOSL1 | GTTCTTTGTAGTCACATATTT |
| sh1-PRMT1 | CCTTAAAGACCAAACTGTTAT |
| sh2-PRMT1 | AGGTTAGCTTATGTTACATAT |
| sh3-PRMT1 | CTTGAAGTGCCTGACTCAATA |
| sh1-CAPS | CCAAGTCAAATACCAAGCAAA |
| sh2-CAPS | CGAGTCATCCGTGGAGGCATTG |
| sh3-CAPS | CGUCAGAUUAUACUGGAAATT |

**Supplementary Table S2.** List of primers used for qRT-PCR in this study

| **Gene** | **Sequence (5’ to 3’)** |
| --- | --- |
| FOSL1-Forward | GGAGGAAGGAACTGACCGACTT |
| FOSL1-Reverse | CTCTAGGCGCTCCTTCTGCTTC |
| PRMT1-Forward | TGCGGTGAAGATCGTCAAAGCC |
| PRMT1-Reverse | GGACTCGTAGAAGAGGCAGTAG |
| CAPS-Forward | GAAGAAAGCGGAGCCTCAGGAA |
| CAPS-Reverse | CGGTCTTGTTCATCGTCACTGG |
| GAPDH-Forward | GTCTCCTCTGACTTCAACAGCG |
| GAPDH-Reverse | ACCACCCTGTTGCTGTAGCCAA |
| SESN3-Forward | GACAGTGACCTGCTATCCTGAG |
| SESN3-Reverse | CCGAGTTATGGCACGAAGAGCA |
| NRCAM-Forward | TGTGGCTGAAGGACAACAGGGA |
| NRCAM-Reverse | AGACGCTGTCCAGAGTGGTGTT |
| PLEKHG1-Forward | ACGGAAGGCTACGATGTTGTGC |
| PLEKHG1-Reverse | CCCAGTTGGTTAGCAAGCTCTG |
| CKB-Forward | GGCAAGCATGAGAAGTTCTCGG |
| CKB-Reverse | ACCAGCTCCACCTCTGAGAAGC |
| MT-ND4L-Forward | CCCTCGTAGTAACAGCCATTCTC |
| MT-ND4L-Reverse | CGACTGTGAGTGCGTTCGTAGT |
| IL13RA2-Forward | GTGGAGTGATAAACAATGCTGGG |
| IL13RA2-Reverse | TGGGTAGGTGTTTGGCTTACGC |
| MICAL2-Forward | TGACAGCCAAGAAGCAGAGCCT |
| MICAL2-Reverse | GGTAGTTGGTGGCAAAGTCTGC |
| ADAMTS1-Forward | GCGTCAATGCTTTCCAACCTGG |
| ADAMTS1-Reverse | GGGATTCTGAGGCTTGTCCATC |
| CEP290-Forward | GGAAGTAGAGTCCCTCAGAATGC |
| CEP290-Reverse | ACCAAGAGCAGTAGCCTCACTC |

**Supplementary Table S3.** List of antibodies used in this study

| **Antibody name** | **Company** | **Catalog number** | **Application** |
| --- | --- | --- | --- |
| FOSL1 | Abcam | ab252421 | WB, IF, IP |
| FOSL1 | Sigma | SAB2108461 | IHC |
| PRMT1 | Proteintech | 11279-1-AP | WB, IHC, IP |
| PRMT1 | Abcam | ab7027 | IF |
| CAPS | Sigma | SAB1411483 | WB |
| CAPS | Sigma | HPA043520 | IHC |
| γ-H2AX | Cell Signaling Technology | 9718 | IF |
| CD31 | Sigma | [SAB5700697](https://www.sigmaaldrich.cn/CN/zh/product/sigma/sab5700697) | IHC |
| ZO-1 | Proteintech | 21773-1-AP | WB, IHC |
| MMP - 2 | Proteintech | 10373-2-AP | WB, IHC |
| N-cadherin | Proteintech | 22018-1-AP | WB, IHC |
| RAD51 | Proteintech | 67024-1-Ig | IF |
| NBS1 | Proteintech | 55025-1-AP | IF |
| Mre11 | Proteintech | 10744-1-AP | IF |
| Ku70 | Proteintech | 10723-1-AP | IF |
| Ku80 | Proteintech | 16389-1-AP | IF |
| 53BP1 | Proteintech | 20002-1-AP | IF |
| H4R3me2a | Proteintech | 39705 | WB |
| MMA | Cell Signaling Technology | 8015 | WB, IP |
| ADMA | Cell Signaling Technology | 13522 | WB, IP |
| PABPN1 | Proteintech | 66807-1-Ig | WB, IP |
| METTL3 | Proteintech | 15073-1-AP | WB |

**Supplementary Table S4.** List of primers used for MeRIP-qPCR analysis.

| **Gene** | **Sequence (5’ to 3’)** |
| --- | --- |
| PARP1-Forward | GAGAGGTAGCCGAGTCACAC |
| PARP1-Reverse | ACCCATCAGCAACTTAGCGG |
| SIRT1-Forward | CGGCTACCGAGGTCCATATAC |
| SIRT1-Reverse | ACAATCTGCCACAGCGTCAT |
| SNAP29-Forward | GACTGACAACCAAAGTGGACAAGC |
| SNAP29-Reverse | AACACATTTTTCCCCCATTGAAACT |
| TSC1-Forward | CTTTCCTCATCGTTCAGCCG |
| TSC1-Reverse | TGTGGTGGTTCAGTTATCAGCC |
| IRF4-Forward | GCCCAGCAGGTTCACAACTA |
| IRF4-Reverse | TGTCACCTGGCAACCATTTTC |
